# Retention-time prediction in comprehensive two-dimensional gas chromatography to aid identification of unknown contaminants

**DOI:** 10.1007/s00216-018-1415-x

**Published:** 2018-10-25

**Authors:** Cathrin Veenaas, Anna Linusson, Peter Haglund

**Affiliations:** Department of Chemistry, Umeå University, 90187 Umeå, Sweden

**Keywords:** GC × GC, Retention-time prediction, Partial least squares (PLS), Federation of local models, Quantitative structure–retention relationship (QSRR), Non-target analysis

## Abstract

Comprehensive two-dimensional (2D) gas chromatography (GC×GC) coupled to mass spectrometry (MS, GC×GC-MS), which enhances selectivity compared to GC-MS analysis, can be used for non-directed analysis (non-target screening) of environmental samples. Additional tools that aid in identifying unknown compounds are needed to handle the large amount of data generated. These tools include retention indices for characterizing relative retention of compounds and prediction of such. In this study, two quantitative structure–retention relationship (QSRR) approaches for prediction of retention times (^1^*t*_R_ and ^2^*t*_R_) and indices (linear retention indices (LRIs) and a new polyethylene glycol–based retention index (PEG-^*2*^*I*)) in GC × GC were explored, and their predictive power compared. In the first method, molecular descriptors combined with partial least squares (PLS) analysis were used to predict times and indices. In the second method, the commercial software package ChromGenius (ACD/Labs), based on a “federation of local models,” was employed. Overall, the PLS approach exhibited better accuracy than the ChromGenius approach. Although average errors for the LRI prediction via ChromGenius were slightly lower, PLS was superior in all other cases. The average deviations between the predicted and the experimental value were 5% and 3% for the ^1^*t*_R_ and LRI, and 5% and 12% for the ^2^*t*_R_ and PEG-^*2*^*I*, respectively. These results are comparable to or better than those reported in previous studies. Finally, the developed model was successfully applied to an independent dataset and led to the discovery of 12 wrongly assigned compounds. The results of the present work represent the first-ever prediction of the PEG-^*2*^*I*.

**Electronic supplementary material:**

The online version of this article (10.1007/s00216-018-1415-x) contains supplementary material, which is available to authorized users.

**Graphical abstract:**
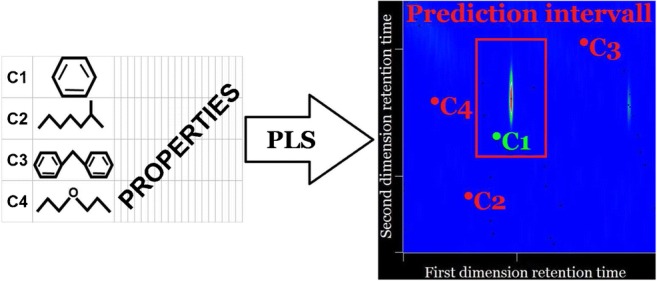
ᅟ

## Introduction

Nowadays, an ever-increasing number of chemicals is being produced and used. More than 100,000 chemicals are used daily [1] and, hence, the need to identify compounds through a non-directed analysis (non-target screening) is great. In addition, an ever-increasing number of compounds are being produced to either aid in developing better manufactured goods or replace pre-existing compounds that have undesirable side effects (e.g., toxicity or persistence). Unknown compounds may be identified via different techniques. Gas chromatography (GC) or liquid chromatography (LC) coupled to mass spectrometry (MS) [2] is typically used to identify these compounds. In addition, comprehensive two-dimensional gas chromatography (GC × GC) has been used to increase the selectivity and separation power, thereby further improving the possibilities of non-target screening. Retention indices (RI) are widely used for the characterization of compounds. Originally, RIs were determined by comparing the relative retention of analytes to nearby eluting *n*-alkanes during an isothermal run [3]. Since then, several improvements and modifications have been realized, including the use of temperature-programmed runs for the calculation of linear retention indices (LRIs) [4, 5]. Moreover, different ways of calculating RIs for GC×GC have been developed [6–11]. Recently, we introduced a new retention index system for GC×GC that was validated for several different column configurations and GC settings [12]. The system uses three short steps to calculate the retention index based on the elution of a series of polyethylene glycols (PEGs). In the present study, we tested different methods and developed different models for the prediction of (i) first-dimension LRIs, (ii) retention index values associated with second-dimension retention (PEG-^*2*^*I*), and (iii) first- and second-dimension retention times.

The prediction of retention times is useful for the characterization and identification of compounds. Different types of models can be used to determine and predict the retention behavior of compounds and characterize their elution pattern. The prediction of GC × GC separations (for example, by using experimental LRIs from single-column temperature-programmed separations [10, 13]) has been extensively investigated. The first-dimension separation in GC×GC depends primarily on the analyte’s vapor pressure, which correlates to its boiling point. However, the second-dimension retention in temperature-programmed GC×GC depends on both the analyte’s polarity and polarizability, and the elution temperature from the first column, which correlates to the first-dimension retention index [10]. Hence, the retention prediction for the second dimension is more complex than the corresponding prediction for the first dimension. Retention-time predictions have been used in several fields of study including proteomics [14, 15], metabolomics [16], or the analysis of organic pollutants using GC-MS or LC-MS techniques [16, 17]. These predictions are applicable to various compounds with different molar masses, polarities, and boiling points [17]. In addition, such predictions can be performed in various ways. These include using thermodynamic properties in mobile and stationary phases in GC [17], a federation of local models approach in combination with physico-chemical properties [18], neural networks [18], and quantitative structure–retention relationships (QSRR) with partial least squares (PLS) [19] to derive an analyte’s retention time or index, respectively, from its structure.

In this study, we focus on two of the aforementioned approaches, QSRR with PLS and the federation of local models. In the PLS approach molecular descriptors, which describe the structure and properties of a molecule via a vector of numbers, are used as variables. The dimensionality in PLS is then reduced by introducing new latent variables (components) that account for maximum variability while at the same time adjusting the latent variables for the response (here, retention time or index). Subsequently, a linear relation between these variables and the response is generated [20]. The federation of local models approach uses a knowledge base, i.e., a large number of compounds (in the case of retention-time predictions) with a known retention time and calculated physico-chemical properties [21]. A new input (compound structure) is then compared to the knowledge base and a limited number of similar entries are selected. These structures are selected using a similarity coefficient, which is calculated using a vector of properties and a distance metric (e.g., the Euclidean distance). These subsets of structures are then used to predict the retention time for the new compound [18].

## Materials and methods

### Overview

In total, four different responses were predicted: the first-dimension retention time (^1^*t*_R_), the first-dimension linear retention index (LRI), the second-dimension retention time (^2^*t*_R_), and the recently established second-dimension retention index that is based on the elution of polyethylene glycols (PEG-^*2*^*I*). Each of these responses was predicted using a separate model.

### Data acquisition

In total, 859 compounds (see Electronic Supplementary Material (ESM), ESM_2) of different chemical classes (e.g., *n*-alkanes, PEGs, pesticides, organophosphates (OPs), fatty acid methyl esters (FAMEs), polycyclic aromatic hydrocarbons (PAHs), polychlorinated dibenzo-p-dioxins and dibenzofurans, bisphenols, polybrominated diphenyl ethers (PBDEs), and all 209 polychlorinated biphenyl (PCB) congeners) were analyzed by GC×GC. This analysis was performed on an Agilent Technologies 6890 gas chromatograph (Palo Alto, CA, USA) coupled to a Pegasus 4D time-of-flight mass spectrometer (TOF MS; Leco Corp., St. Joseph, MI, USA). For GC×GC analysis, a secondary oven and a quad-jet dual stage modulator were located in the main GC oven. A 30-m non-polar Rtx-5sil ms column (Restek, Bellefonte, PA, USA) was used for the first-dimension separation and a 1.6-m semi-polar BPX50 column (SGE, Trajan Scientific Europe Ltd., Crownhill, Milton Keynes, UK) was used for the second-dimension separation. This coupling corresponded to the most commonly used combination of stationary phases [22, 23] in environmental analysis and for both columns the internal diameter (i.d.) and film thickness were 0.25 mm and 0.25 μm, respectively. A deactivated capillary (0.25 mm i.d.) was used in the transfer line, which was held at a temperature of 350 °C. The split/split-less injector was operated in split mode (temperature 280 °C, split ratio 1:10) to reduce the influence of the injection solvent on the retention times. The temperature program for the first oven consisted of heating at 35 °C for 0.2 min, increasing the temperature at a rate of 5 °C/min to 310 °C, and holding for 12 min. The secondary oven had an offset of +30 °C relative to the first oven and the modulator had an offset of + 20 °C relative to the secondary oven. A modulation period, hot jet duration, and cold jet duration of 5 s, 0.61 s, and 1.89 s, respectively, were employed. Helium (flow rate 1 mL/min) was used as the carrier gas. Electron ionization (EI) was performed at an electron energy and an ion source temperature of 70 eV and 280 °C, respectively. An MS acquisition rate of 100 spectra/s was used for all runs and data were collected for *m*/*z* ranging from 29 to 750. Data were acquired and processed using the ChromaTOF software (version 4.50; Leco Corp.).

### Molecular descriptors

Molecular descriptors (ESM_1, Table S1) were calculated for all compounds using the Molecular Operating Environment (MOE, version 2016.08, Chemical Computing Group Inc., Montreal, QC, Canada) software (104 2D physico-chemical descriptors) and the Percepta software (29 2D physico-chemical descriptors) with the Absolv add-on module (to calculate Abraham solvation parameters) from ACD/Labs (Advanced Chemistry Development UK Ltd., Bracknell, England). In addition, to compensate for the size dependency of some properties (e.g., lipophilicity, logK_OW_) additional descriptors were introduced, in which the properties were normalized to the weight of the compound. Finally, for each descriptor, a manual transformation (see ESM_1 Tables S2-S5 for types of transformations) of the data was performed to determine whether this would create a more linear relationship between the descriptor and the response. This yielded 20 and three additional transformed descriptors for the first-dimension and second-dimension models, respectively.

### Calculations and data pre-treatment

First-dimension LRIs and second-dimension PEG-^*2*^*I* values were calculated as described in references [4, 12], respectively. The compounds were divided into three sets of data: a training set, a test set, and an external validation set. Of the 209 PCBs, all except two per chlorination level were added to the external validation set to avoid “over-training” the models for PCB predictions. Afterward, the remaining compounds were systematically divided into the three sets. The data were divided by performing a principle component analysis (PCA) [24, 25] with five components on all the compounds using the MOE molecular descriptors. As a result, the first, second, and third components explain 43%, 20%, and 10% of the variation, respectively. The last two components explain less than 10% each (6% and 3%). The compounds were sorted in ascending order of the first component scores. Every fifth value was assigned to the training set, every eighth to the test set, and every ninth to the external validation set. The remaining data were then sorted in ascending order of the second component scores and the procedure was repeated. These steps were repeated for the first four components. The remaining compounds were sorted into the three datasets randomly. After the division was completed, PCA and PLS score plots were generated and the training and test sets were compared to ensure that they cover the same space.

The training set was used to create the models. Each model was then optimized with regard to different parameters (described in subsequent sections) using the test set. The predictive power of the model was compared after each step. The final model was validated using the external validation set. For each of the two approaches described below, four models were created, optimized, and validated, namely the (i) ^1^*t*_R_, (ii) LRI, (iii) ^2^*t*_R_, and (iv) PEG-^*2*^*I* models.

The models were evaluated and compared via the root-mean-square error of prediction (RMSE_P_), which was determined from:

$$ {RMSE}_P=\sqrt{\frac{1}{N-1}\sum {\left({y}_{\mathrm{obs}}-{y}_{\mathrm{pred}}\right)}^2} $$

where *N*: number of data points and *y*_obs_ and *y*_pred_: experimental (observed) and predicted values (here retention times and indices), respectively. The *y*_pred_ for the RMSE_P_ was obtained by predicting compounds from the test set or external validation set during model optimization and validation, respectively.

### Partial least squares

The SIMCA software (version 14, Umetrics AB, Umeå, Sweden) was used to create the PLS models. The molecular descriptors along with the responses (^1^*t*_R_, LRI, ^2^*t*_R_, and PEG-^*2*^*I*) were imported (ESM_2) and the data were then centered and scaled to unit variance. Separate models were created for each response using the training set. The number of PLS components was determined using cross-validation (seven groups) and the models were optimized using the test set. Various factors were assessed including the stepwise increase in the number of descriptors, automatic transformation of variables through SIMCA, removal of all molecular descriptors with uncertainties larger than their contribution, removal of all molecular descriptors of low importance (< 1), stepwise (10 at a time) removal of descriptors characterized by high uncertainty and low importance, and creation of local models. The stepwise removal of descriptors was performed until all descriptors that remained had a low uncertainty and high importance. For each new model, the number of latent variables (components) was determined by optimizing the predictive power and errors.

### “Federation of local models”

The federation of local models approach was performed in the ChromGenius software package (version 2017.1.3, ACD/Labs). The structures of all compounds and the respective responses (^1^*t*_R_, LRI, ^2^*t*_R_, and PEG-^*2*^*I*) were imported using an .sdf file (ESM_3). As previously mentioned, separate models were created for each response. The ^1^*t*_R_ values were imported as minutes while keeping ^2^*t*_R_ in seconds and choosing retention time in minutes in ChromGenius. Otherwise, there would have been an unacceptable loss of time-resolution (ChromGenius handles a limited number of decimals). This does not change the variability in the data and should, thus, not affect the accuracy of the predictions. The effect of the following factors on the RMSE_P_ was evaluated: using the dice coefficient and Euclidian distance for the similarity search, changing the number of similar compounds (20 and 25) used for the prediction, excluding Abraham parameters, excluding all parameters except for the Abraham parameters, and changing the number of compounds per parameter (three or four) included in the equation for the retention-time calculation.

### Validation

The retention times and indices for compounds from the external validation set were predicted using optimized versions of both the PLS and ChromGenius models. The RMSE_P_ was calculated for each model response and the results from the prediction of the test set and external validation set were compared. In addition, the two methods, PLS and ChromGenius, were compared.

### Benchmarking

To evaluate the quality of the models two approaches were performed according to reference [26]. Simple linear models using only one basic descriptor (boiling point for the first-dimension and logK_OW_ and the logK_OW_ normalized to the weight of the compound for the second-dimension models) were generated for all four responses. The predictive power of these reference models was compared with those of the previously developed models to see if advanced models improve the prediction. If a comparable predictive capability was revealed, the PLS and ChromGenius would be considered overly complicated, and a simple one-parameter model could be used instead. In addition, for each compound in the external validation set the measured value was compared to the average value and the RMSE_P_ was calculated using the difference of those two. The RMSE_P_ values of these approaches are expected to be considerably higher than those of the developed models if good models were achieved.

## Results and discussion

### PLS modeling

The effect of each optimization step on the RMSE_P_ of all four responses is shown in Table 1. Use of all the MOE, Percepta, and manually transformed descriptors yielded the best result for the first-dimension models (both ^1^*t*_R_ and LRI). However, the best results for the second-dimension prediction models were realized by using these three sets and all descriptors normalized to the molecular weight. The second-dimension separation is based on the polarity and polarizability of a compound. The polarizability may be size dependent, and hence, the inclusion of descriptors normalized to the weight improved the model. Additional optimization attempts yielded no further improvement. Therefore, the addition of further descriptors improves, in general, the predictive power of the models.

**Table 1:** Root-mean-square error of prediction (RMSE_P_) for PLS models of varying complexities using the test set and the lowest and highest measured values of each response

Descriptors used in model	RMSE_P_*			
	^1^t_R_ (s)	LRI	^2^t_R_ (s)	PEG-^*2*^*I*
MOE only	142 (6)	116 (8)	0.37 (8)	15.3 (8)
MOE and Percepta	118 (7)	104 (6)	0.33 (6)	13.2 (7)
MOE, Percepta, and manually transformed descriptors	*112* (*6*)	*101* (*7*)	0.33 (6)	13.3 (7)
MOE, Percepta, manually transformed, and normalized-to-weight descriptors	119 (7)	106 (7)	*0.30* (*7*)	*12.5* (*8*)
All, auto-transformed	114 (9)	107 (8)	0.32 (7)	20.2 (8)
All, except those with high uncertainty	123 (6)	114 (6)	0.32 (7)	14.4 (7)
All, except those of low importance	132 (6)	108 (8)	0.30 (7)	14.2 (8)
All, except those with high uncertainty and low importance (stepwise removal)	145 (4)	–	0.41 (2)	–
Lowest measured value	270	808	1.68	0
Highest measured value	3325	3413	6.62	215.1

* The number of PLS components is shown in parentheses. The italicized values indicate the model with the lowest RMSE_P_ for each response, respectively. ^1^t_R_, LRI, ^2^t_R_, and PEG-^*2*^*I* are the first-dimension retention time, linear retention index, second-dimension retention time, and polyethylene glycol–based second-dimension retention index, respectively

Further improvement was attempted by developing local models as an alternative to a single model for all compounds (global model). The compounds were grouped based on a PCA score plot. The hypothesis was that compounds exhibiting similar behavior would cluster in these plots and therefore be well-suited for consideration by a local model. Three groups were defined, based on the PCA. An additional group was developed for fluorinated compounds, which were associated with the largest errors in the predictions of each model. Hence, the resulting four groups are (as indicated in ESM_2): fluorinated compounds (group 1), chlorinated and brominated compounds (group 2), non-polar compounds with (long) carbon-based chains (including, for example, alkanes, PEGs, glymes, and FAMEs; group 3), and all remaining compounds (group 4). Compared with the best global model (i.e., lowest RMSE_P_ from Table 1), the locally generated models provided significantly better predictions only in the case of group 3 (i.e., compounds with long carbon-based chains). The RMSE_P_ values for the ^1^*t*_R_ and LRI models improved from 64 to 14 s and 49 to 5, respectively. Similarly, the ^2^*t*_R_ and PEG-^*2*^*I* models improved from 0.21 to 0.02 s and 7.6 to 0.9, respectively. The improvement realized for the second dimension was considerably larger than that realized for the first dimension. Furthermore, the uncertainty associated with prediction of the second-dimension retention times and indices is, in general, higher than that of the corresponding first-dimension values, and hence, the improvement through local models is a great advantage here. However, low or no improvement was realized for the remaining models, and hence, further consideration was deemed unnecessary.

### ChromGenius models

Varying results were obtained for the ChromGenius models, where a clear trend for the first- or second-dimension models was lacking. For example, similarity calculations based on the dice coefficient and the Euclidean distance yielded the best results for the first- and second-dimension models, respectively (Table 2). For the LRI model, the best results were obtained when the number of compounds for the local model was limited to 20. However, the best results for the other three models were obtained when 25 compounds were considered.

**Table 2:** Root-mean-square error of prediction (RMSE_P_) for each model optimization step with ChromGenius using the test set and lowest and highest measured values of each response

Model settings	RMSE_P_*			
	^1^t_R_ (s)	LRI	^2^t_R_ (s)	PEG-^*2*^*I*
Dice coefficient (25 compounds)	**159**	93	0.28	14.6
Dice coefficient (20 compounds)	160	***84***	0.32	17.2
Euclidian distance (25 compounds)	196	105	**0.28**	***14.5***
Euclidian distance (20 compounds)	204	98	0.29	15.5
Best model setting, no Abraham parameters	176	155	0.34	17.5
Best model setting, only Abraham parameters	*158*	153	0.34	22.6
Three instead of four molecules used per parameter	160	135	*0.26*	18.1
Lowest measured value	270	808	1.68	0
Highest measured value	3325	3413	6.62	215.1

* The italic and the bold values indicate the results of the final model and the best model from the first step, respectively. ^1^t_R_, LRI, ^2^t_R_, and PEG-^*2*^*I* are the first-dimension retention time, linear retention index, second-dimension retention time and polyethylene glycol–based second-dimension retention index, respectively

The possibility of further model improvement was investigated by excluding (i) the Abraham parameters and (ii) all other parameters and using the Abraham parameters only. The results revealed that omitting these parameters had no effect on improving the models. However, using only Abraham parameters (and omitting all other physico-chemical properties) resulted in an improved model for the ^1^*t*_R_. Moreover, the ^2^*t*_R_ model improved when three (rather than four) compounds per parameter were used in the calculation of the final predicted value.

The results of the best model corresponding to each variable (^1^*t*_R_ and ^2^*t*_R_ as well as LRI and PEG-^*2*^*I*) are written in italic in Table 2. Some models exhibit similar RMSE_P_ values (e.g., 158 s vs. 159 s and 160 s for the ^1^*t*_R_ model). Using the given data collected, it is not possible to assess whether this difference is significant since no replicate models were performed using different datasets. Hence, it must be considered that those values could be equal. In this case, the final models could be chosen in a way that the ^1^*t*_R_ model and LRI model and the ^2^*t*_R_ model and PEG-^*2*^*I* model, respectively, are calculated using the same parameters. For example, both first-dimension models would be obtained using the dice coefficient model with 20 compounds and the second-dimension models would be created using the Euclidian distance model with 25 compounds (Table 2), including four parameters in the calculation of the responses in both cases. In this case, however, we chose the models that revealed the lowest prediction error value.

### Validation and comparison

**Fig. 1:**
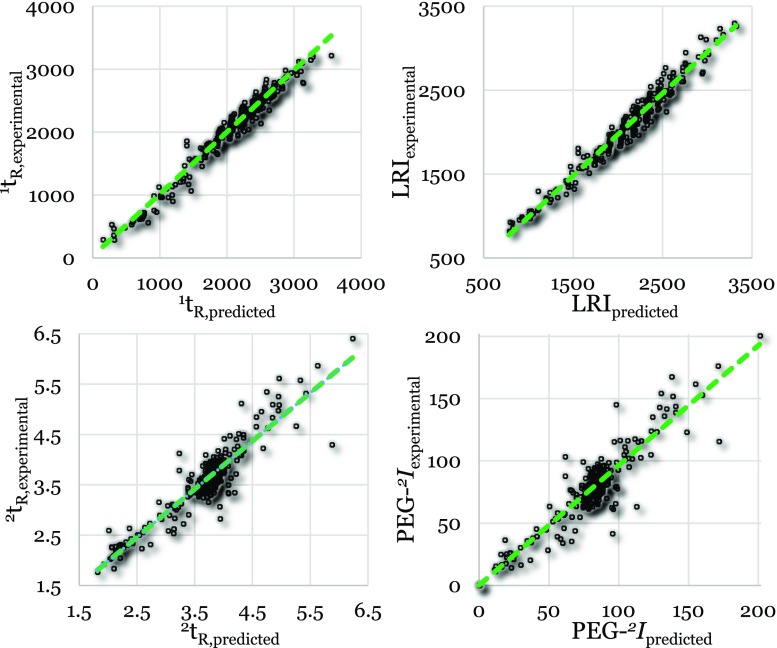
Predicted vs. experimental values for the external validation set using PLS. ^1^*t*_R_, LRI, ^2^*t*_R_, and PEG-^*2*^I are the first-dimension retention time, linear retention index, second-dimension retention time, and polyethylene glycol-based second-dimension retention index, respectively

**Fig. 2:**
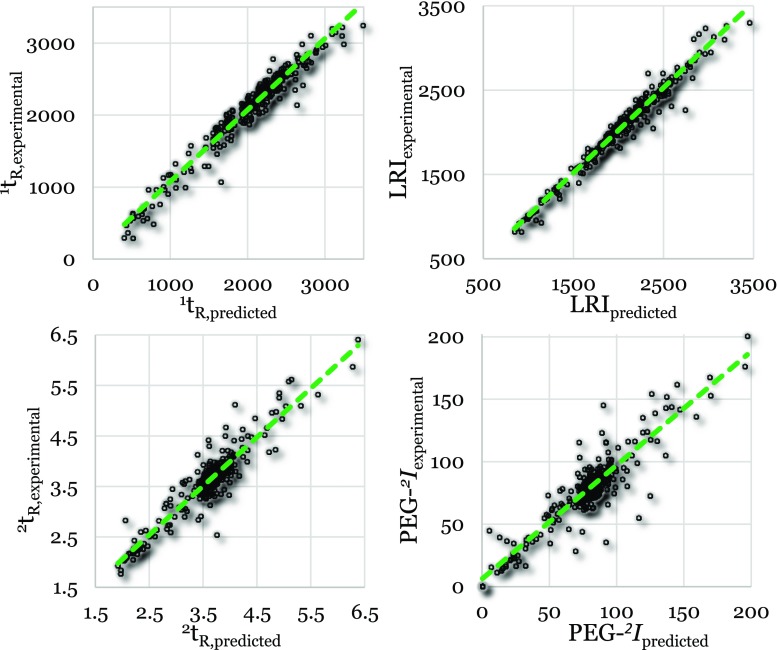
Predicted vs. experimental values for the external validation set using ChromGenius. ^1^*t*_R_, LRI, ^2^*t*_R_, and PEG-^*2*^I are the first-dimension retention time, linear retention index, second-dimension retention time, and polyethylene glycol-based second-dimension retention index, respectively

The final models were validated by predicting the external validation set using the aforementioned best models. A table containing all values can be found in the ESM (ESM_1, Table S6). In general, the first-dimension models performed better than the second-dimension models, independent of the technique used for the prediction, as shown in Figs. 1 and 2. As previously explained in the “Introduction” section, the first-dimension separation occurs independently of the second-dimension separation, while the second-dimension separation is affected by the first-dimension retention (the ^1^*t*_R_ determines the temperature at which an analyte enters the secondary column). However, the second-dimension separation depends primarily on selective interactions with the stationary phase that depend on the type of functional groups or moieties comprising the analytes. The analyte may have structural domains that contribute to many physico-chemical properties and structural descriptors but contribute nothing to the second-dimension retention. For example, all *n*-alkanols will have similar ^2^*t*_R_, but very different log K_OW_ values, which will be strongly correlated with the alkane-chain length. Therefore, prediction of the second-dimension separation is more complex than prediction of the first-dimension separation, and hence, the corresponding errors are higher.

The predictions performed using the external validation set were in all cases similar to or more accurate than the predictions performed using the test set (see Table 3). Hence, the developed models are valid. The relative deviations of the predicted values from the experimental values for the first-dimension PLS and ChromGenius models (Table 3) are comparable (average: 5% vs. 6% and 4% vs. 3% for ^1^*t*_R_ and LRI, respectively). However, the calculated errors (i.e., RMSE_P_) associated with the ^1^*t*_R_ are higher for the ChromGenius model than for the PLS model. For the LRI model, ChromGenius produced slightly better RMSE_P_ values than the PLS model. However, LRI prediction accuracies suggest that the PLS model is superior to the ChromGenius model (85–114% and 86–123% for the PLS model and ChromGenius, respectively). Using ChromGenius, Dossin et al. [18] realized accuracies of 86–126%, which are similar to the ChromGenius results obtained here. However, the PLS model developed here yields better results than those reported by Dossin and coworkers. Furthermore, the group contribution model (GCM) developed by the National Institute of Standards and Technology (NIST) [27] resulted in an average deviation of 4.4% for the prediction of LRI values. As previously mentioned, the PLS- and ChromGenius-based models developed in the present work resulted in errors of 4% and 3%, respectively. Therefore, these models are equally good or slightly better than the model developed at NIST [27]. In general, the average deviation of the GCM is relatively low for compounds with few or no functional groups, e.g., 39 RI units for alkanes [27, 28]. Low errors were also observed for our local models corresponding to long-carbon chain compounds (average deviation of 11 RI units). However, the performance of the GCM for compound groups with more and diverse functional groups (than these compounds) is less good. The average deviation for all modeled compounds (almost 23,000) is 70; and for multifunctional compounds it is 88, which is similar to the values in Table 3.

**Table 3:** Prediction errors (RMSE_P_) and average relative deviation of the predicted value from the experimental value for all four models using PLS and ChromGenius and the test set and external validation set, respectively

	External validation set prediction *				Test set prediction *			
	^1^t_R_	LRI	^2^t_R_	PEG-^*2*^*I*	^1^t_R_	LRI	^2^t_R_	PEG-^*2*^*I*
PLS								
Average relative deviation	5%	4%	5%	12%	7%	5%	6%	16%
Average deviation	80 s	74	0.19 s	7.8	85 s	74	0.20 s	8.2
RMSE_P_	109 s	95	0.27 s	11.3	121 s	105	0.29 s	12.2
ChromGenius								
Average relative deviation	6%	3%	4%	12%	9%	3%	5%	17%
Average deviation	115 s	60	0.16 s	7.8	124 s	57	0.17 s	9.2
RMSE_P_	143 s	85	0.23 s	11.8	158 s	84	0.26 s	14.5

* ^1^t_R_, LRI, ^2^t_R_, and PEG-^*2*^*I* are the first-dimension retention time, linear retention index, second-dimension retention time, and polyethylene glycol–based second-dimension retention index, respectively. The results for the PEG-^*2*^*I* models include compounds that were extrapolated due to a narrow PEG range

For the second-dimension models, the relative deviations of the PLS and ChromGenius models are similar, as in the case of the first-dimension models. The corresponding RMSE_P_ values for the ^2^*t*_R_ and PEG-^*2*^*I* are slightly better using ChromGenius and PLS, respectively. For both types of second-dimension models, the variations associated with the index model were larger than those of the retention-time model as can be seen in the respective figures as well as calculated relative deviations (Table 3, Figs. 1 and 2). This increased error resulted most likely from the additional variation associated with the PEG-^*2*^*I* calculations. D’Archivio et al. [19] performed a GC×GC retention-time prediction study where PCBs were considered. In that work, relative deviations of 1.6–2.9% were realized when different modeling approaches (multi-linear regression, artificial neural networks, and PLS) were used to predict the ^2^*t*_R_. These relative deviations are slightly better than the values obtained in this study (5% and 4%). The relative deviation decreased to 3%, however, when only PCBs were predicted and was, therefore, comparable to the value obtained by D’Archivio et al. However, the models used in this study were built using a considerably larger selection of compounds with more diverse chemical properties than the set of compounds considered in that work. Therefore, improved accuracy of the PLS and ChromGenius models employed in the present study is expected when only PCBs are considered during the model building. To reduce the error associated with the PEG-^*2*^*I* calculation other compounds could be chosen as indexing references. Preliminary results have shown that selected PAHs (i.e., 1-methylnaphthalene, acenaphthylene, anthracene, fluoranthene, benz(a)anthracene, benzo(e)pyrene, and benzo(g,h,i)perylene) form a straight line in the GC×GC chromatogram, similar to the PEGs. Their polarizabilities and retention increases according to the number of fused aromatic and non-aromatic rings (2 aromatic rings, 2 aromatic and 1 non-aromatic rings, 3 aromatic rings, 3 aromatic and 1 non-aromatic rings, 4 aromatic rings, 5 aromatic rings and 6 aromatic rings, respectively). Since these compounds are less polar, they are easier to analyze than the highly polar PEGs due to reduced binding to active sites in the GC system and, hence, reduced peak tailing.

### Quality assurance

The linear regression models, i.e., reference models for benchmarking, using boiling points and lipophilicity (logK_OW_ and logK_OW_ / molecular weight), resulted in RMSE_P_ values that were almost double and more than double those of the PLS and ChromGenius models for the first and second dimensions, respectively. Hence, the PLS and the ChromGenius models are better than a simple model based on a single descriptor. Furthermore, all models can give a better prediction than the average. The RMSE_P_ values were four to five times higher when using the average for the first-dimension models and more than double when using the average for the second-dimension models compared to the final developed models.

### Detailed evaluation of the PLS models

Evaluations of the loading scatter plot, coefficients plot, and variable importance plot within the SIMCA software for PLS modeling show that the boiling point accounts for one of the largest contributions in the first-dimension models. This is unsurprising as the separation on a non-polar column in the first dimension is, in principle, based on the volatility of the compound being considered. In connection with this, the separation in both dimensions is largely dependent on the partitioning coefficient between gas phase and hexadecane (L), one of the Abraham solvation parameters. Considering that the separation technique used was gas chromatography a larger contribution of this specific partition coefficient was expected. Other parameters, such as the surface tension and index of refraction, account for large contributions in the second-dimension models. The index of refraction is linked to the polarizability of a compound [29] which is one of the factors that influence the separation on the second column in GC×GC. Therefore, when using a semi-polar secondary column, the larger contribution of this factor to the second-dimension models (compared with other descriptors) is understandable. Accordingly, the polarity/polarizability parameter (S), another Abraham parameter, shows a large contribution to the second-dimension models. The variable importance for all descriptors in the final models is given in the ESM (ESM_1, Tables S2-S5).

Evaluation of specific compounds revealed that the early eluting compounds exhibit larger relative variations than the late-eluting compounds. This is unsurprising as small differences have a relatively large impact on the prediction at low retention times or indices. In addition, early compounds might be more affected by the injection process and the initial isothermal part, although short, of the temperature program. As previously mentioned, the error obtained for fluorinated compounds was larger than that obtained for other compounds. The reason for this larger error is unclear, but may have resulted from the fact that these compounds were underrepresented in the data used here. In addition, fluorine has the highest electronegativity of all elements and electronegativity plays a role for gas chromatographic retention [30]. Compounds with high electronegativity may form charge-transfer complexes with phenyl groups in the stationary phase and thereby be retained. Consequently, even non-polar halogenated compounds have relatively long second-dimension retention times and indices. To improve the predictive power for fluorinated compounds, the number of fluorinated compounds included in the model-building process (i.e., the training of the model) could be increased. In addition, 1,4-phenylenediamine exhibited a higher deviation in the second-dimension PLS models, respectively, most likely due to its high proportion of functional groups (two amino groups in a small molecule) that strongly interact with the phenyl groups of the stationary phase.

As a final improvement attempt, especially targeting the second-dimension predictions, 153 molecular descriptors representing functional groups were obtained through the Dragon software (version 6.0; Talete s.r.l., Milano, Italy) and, subsequently, included in the modeling. Only a small improvement was obtained for the ^1^*t*_R_ model, reducing the RMSE_P_ for the test set prediction from 112 to 105 s when including Dragon descriptors with a variable importance above 0.5. The importance of the functional group descriptors was, thus, rather low, possibly because the properties of functional groups were already captured by other descriptors. Overall, this result shows that including more descriptors can lead to improved predictions. However, in this case, the improvement was regarded as too small to be worth the effort. It would also add unnecessary complexity to the model.

### Retention time vs. retention index models

The performance of the retention-time models was compared with those of the respective retention-index models (^1^*t*_R_ vs. LRI and ^2^*t*_R_ vs PEG-^*2*^*I*) using both methods, PLS and ChromGenius, respectively. For this comparison, the RMSE_P_ values obtained through the validation of the models were divided by the overall span of the values. The results showed that, for the first-dimension model obtained via ChromGenius, the LRI model yielded better results than the retention-time model. The same relative errors were obtained in all other cases. Since RIs are relative values that are comparable across different instruments and settings while retention times are absolute values that are instrument and setting specific, the use of retention index prediction can be advantageous over retention-time prediction. The ESM includes the data collected for this study (an Excel table for the PLS data and SD files for the ChromGenius models) for the retention times as well as indices which can be used to generate prediction models.

### Application of retention-time prediction models

As discussed in the “Introduction” section, retention-time and retention-index predictions can aid in the identification of unknown compounds in non-target screening studies. Even using GC high-resolution MS, it is often difficult to (tentatively) identify compounds in a complex mixture. Structurally similar compounds generally yield similar mass spectra. Hence, the here-developed models can be used as a tool for the identification of unknown compounds by helping to distinguish among many possible candidate structures or by identifying wrongly assigned structures. Candidates with non-matching structure and elution times can be eliminated. Complementary use of retention index and MS spectral information can greatly reduce the risk of reporting false positive findings and increase the chance to propose correct structures for unknowns.

A candidate structure is deemed incorrect if the predicted retention time or index lies outside the given range of error associated with the experimental value of the unknown compound. In practice, a concrete measure for the range of error (for example, the 95-percentile) is needed. The 95-percentile is easy to understand and gives a clear idea of the likelihood of introducing an error. The 95-percentiles for the prediction errors associated with the here-developed models are listed in Table 4. In general, the 95%-confidence intervals obtained from the NIST group contribution model [28] for the prediction of LRIs are relatively low for compounds with few or no functional groups. Low errors were also observed for the local models corresponding to long-carbon chain compounds. However, other compound groups with more and diverse functional groups (than these compounds) have higher 95%-confidence intervals than the 95-percentiles obtained here as error ranges. To decrease the error of predictions, the model results of the two approaches (PLS and ChromGenius) can be combined by taking the average of both predictions. The values for the average of both model types given in Table 4 are, without exception, lower than those of the individual models.

**Table 4:** 95-percentiles defining the range of error associated with the prediction of each final model

	^1^t_R_ (s)	LRI	^2^t_R_ (s)	PEG-^*2*^*I*
PLS	214	189	0.53	21.0
ChromGenius	258	160	0.48	23.6
Average	195	140	0.41	19.7

^1^t_R_, LRI, ^2^t_R_, and PEG-^*2*^*I* are the first-dimension retention time, linear retention index, second-dimension retention time, and polyethylene glycol–based second-dimension retention index, respectively

Applying this retention time or index-prediction procedure will help to reduce the list of possible candidate structures. However, one possible risk is that new compounds may lie outside the model domain. Therefore, the similarity between the new compound and the compounds used to build the model must be determined. This similarity can be determined through a PCA analysis. The location of the compound of interest (i.e., the new/predicted compound) with respect to the training set in a score scatter plot can then be assessed. The model can still be used for compounds lying outside the model domain, but higher errors, than those associated with compounds lying inside the domain, should be expected. Notably, all compounds included in the external validation set were well within the model domain of the training set.

In theory, when using RIs, the calculated index values should be comparable across instruments and instrument configurations. Therefore, once a model is created for the retention index (first-dimension LRIs as well as second-dimension PEG-^*2*^*I* values) that model can be used to predict RIs for new compounds. Retention times are, however, absolute values and will vary when parameters, such as the settings in the instrument or column lengths, are changed. Hence, new models must be established when conditions are changed. Data from a previously analyzed house dust sample, generated as part of an interlaboratory comparison study, was used to test the applicability of the retention predictions. The sample was analyzed more than a year prior to the current study, using the same stationary phases, but different column dimensions. The house dust contained a range of PEGs and an alkane standard had been run in parallel to the sample. Thus, first- and second-dimension RIs (LRI and PEG-^*2*^*I*) could easily be calculated for all identified compounds. The PLS models were used to predict RIs for the identified compounds and the results were then compared to find compounds that deviated more than expected (Table 4) from the experimental data. In addition, plots of the predicted vs. measured indices were used to provide insight into the (qualitative) structure–retention relationships. After scrutiny of the results, it was concluded that 12 of the 500 compounds that had been tentatively identified in the house dust were likely incorrectly assigned. Two of these, originally wrongly identified as 1,4,7,10,13,16,19-heptaoxa-2-cyclo-heneicosanone and 3,3-dimethyl-(3H)-indazole, could be reassigned to hexaethylene glycol (PEG-6) and α-methylstyrene, respectively. PEG-6 was confirmed to be present in the dust by other participants in the interlaboratory study and the α-methylstyrene peak exhibited a spectrum very similar to the NIST reference spectrum (but was not confirmed with a standard).

Notably, the house dust data had been carefully curated prior to reporting of the data (incl. comparison to NIST retention index data). A dataset that would not have been previously evaluated would, most likely, have contained more misassignments. The 95-percentiles of the deviation between the measured and predicted value of all compounds that were assumed to be correctly identified were 226 and 23.7 for the LRI and PEG-^*2*^*I* predictions, respectively. Those values are deviating 20% and 13%, respectively, from the 95-percentiles of the external validation set (Table 4), which shows good comparability between the two uncertainty estimates. The application of the prediction method to this dataset shows that the retention time/index prediction models can accommodate differences among chromatographic systems can aid in the discovery of false positives and (sometimes) can be used to correct misassignments.

## Conclusion

The PLS model seemed to produce slightly better results (i.e., models with slightly lower prediction errors) in all cases, except for the LRI model, compared with the ChromGenius model. The ChromGenius software suffers from the drawback that an assessment of the applicability domain, as it was suggested above using a PCA, is impossible. The PLS modeling approach is therefore preferred to the use of ChromGenius. In addition, the possibility of improving the PLS predictions by adding more descriptors (for example 3D descriptors) is given. Those could, for instance, include semi-empirical electronic property and charge distribution descriptors. However, some pre-knowledge about the software and PLS modeling (in general) is required, whereas the use of the ChromGenius software is relatively simple. If only first-dimension retention times or indices need to be predicted, the use of a simple Abraham parameter model may be considered. Although less precise, such models would be very easy to generate. In addition to the here-presented model types, other model types that can account for larger degrees of nonlinearity as, for example, machine learning algorithms (e.g., artificial neural networks (ANNs) or support vector machines (SVM)), can be tested to improve models.

## Electronic supplementary material

ESM 1 (PDF 978 kb)

ESM 2 (XLSX 2981 kb)

ESM 3 (7Z 117 kb)
