# Supplementary material for: Retention-time prediction in comprehensive two-dimensional gas chromatography to aid identification of unknown contaminants
Source: Anal Bioanal Chem. 2018 Oct 25;410(30):7931–41. doi: 10.1007/s00216-018-1415-x (PMC6244764; doi:10.1007/s00216-018-1415-x)
Supplement: Supplementary file 1 — (PDF 978 kb) [file 216_2018_1415_MOESM1_ESM.pdf]

Analytical and Bioanalytical Chemistry

Electronic Supplementary Material

Retention-time prediction in comprehensive two-dimensional gas chromatography to aid identification of unknown contaminants

Cathrin Veenaas, Anna Linusson, Peter Haglund

Additional files available under 10.1007/s00216-018-1415-x

Table S1 Molecular Descriptors

Var ID (Primary) *,**	Description
a_acc	Number of hydrogen bond acceptor atoms (not counting acidic atoms but counting atoms that are both hydrogen bond donors and acceptors such as -OH).
a_aro	Number of aromatic atoms.
a_count	Number of atoms (including implicit hydrogens). This is calculated as the sum of $(1 + h_i)$ over all non-trivial atoms i.
a_don	Number of hydrogen bond donor atoms (not counting basic atoms but counting atoms that are both hydrogen bond donors and acceptors such as -OH).
a_donacc	Number of hydrogen bond donor plus number of hydrogen bond acceptor atoms.
a_heavy	Number of heavy atoms $\#\{Z_i \mid Z_i > 1\}$.
a_hyd	Number of hydrophobic atoms.
a_nBr	Number of bromine atoms: $\#\{Z_i \mid Z_i = 35\}$.
a_nC	Number of carbon atoms: $\#\{Z_i \mid Z_i = 6\}$.
a_nCl	Number of chlorine atoms: $\#\{Z_i \mid Z_i = 17\}$.
a_nF	Number of fluorine atoms: $\#\{Z_i \mid Z_i = 9\}$.
a_nH	Number of hydrogen atoms (including implicit hydrogens). This is calculated as the sum of hi over all non-trivial atoms i plus the number of non-trivial hydrogen atoms.
a_nI	Number of iodine atoms: $\#\{Z_i \mid Z_i = 53\}$.
a_nN	Number of nitrogen atoms: $\#\{Z_i \mid Z_i = 7\}$.
a_nO	Number of oxygen atoms: $\#\{Z_i \mid Z_i = 8\}$.
a_nP	Number of phosphorus atoms: $\#\{Z_i \mid Z_i = 15\}$.
a_nS	Number of sulfur atoms: $\#\{Z_i \mid Z_i = 16\}$.
AlphaH2 (A)	Overall solute H-Bonding acidity
apol	Sum of the atomic polarizabilities (including implicit hydrogens) with polarizabilities taken from [CRC 1994].
b_1rotN	Number of rotatable single bonds. Conjugated single bonds are not included (e.g. ester and peptide bonds).
b_1rotR	Fraction of rotatable single bonds: b_1rotN divided by b_heavy.
b_ar	Number of aromatic bonds.
b_count	Number of bonds (including implicit hydrogens). This is calculated as the sum of $(d_i/2 + h_i)$ over all non-trivial atoms i.
b_double	Number of double bonds. Aromatic bonds are not considered to be double bonds.
b_heavy	Number of bonds between heavy atoms.
b_max1len	Length of the longest single bond chain.
b_rotN	Number of rotatable bonds. A bond is rotatable if it has order 1, is not in a ring, and has at least two heavy neighbors.
b_rotR	Fraction of rotatable bonds: b_rotN divided by b_heavy.
b_single	Number of single bonds (including implicit hydrogens). Aromatic bonds are not considered to be single bonds.
b_triple	Number of triple bonds. Aromatic bonds are not considered to be triple bonds.
balabanJ	Balaban's connectivity topological index [Balaban 1982].
BetaH2 (B)	Overall solute H-Bonding basicity
BetaO (Bo)	Overall solute H-Bonding basicity
BP (Pressure (mmHg) = 760,00)	Boiling point
bpol	Sum of the absolute value of the difference between atomic polarizabilities of all bonded atoms in the molecule (including implicit hydrogens) with polarizabilities taken from [CRC 1994].
C Ratio	C Ratio
chi0	Atomic connectivity index (order 0) from [Hall 1991] and [Hall 1977]. This is calculated as the sum of $1/\mathrm{sqrt}(d_i)$ over all heavy atoms i with $d_i > 0$.
chi0_C	Carbon connectivity index (order 0). This is calculated as the sum of $1/\mathrm{sqrt}(d_i)$ over all carbon atoms i with $d_i > 0$.
chi0v	Atomic valence connectivity index (order 0) from [Hall 1991] and[Hall 1977]. This is calculated as the sum of $1/\mathrm{sqrt}(v_i)$ over all heavy atoms i with $v_i > 0$.
chi0v_C	Carbon valence connectivity index (order 0). This is calculated as the sum of $1/\mathrm{sqrt}(v_i)$ over all carbon atoms i with $v_i > 0$.
chi1	Atomic connectivity index (order 1) from [Hall 1991] and [Hall 1977]. This is calculated as the sum of $1/\mathrm{sqrt}(d_i d_j)$ over all bonds between heavy atoms i and j where $i < j$.
chi1_C	Carbon connectivity index (order 1). This is calculated as the sum of $1/\mathrm{sqrt}(d_i d_j)$ over all bonds between carbon atoms i and j where $i < j$.
chi1v	Atomic valence connectivity index (order 1) from [Hall 1991] and [Hall 1977]. This is calculated as the sum of $1/\mathrm{sqrt}(v_i v_j)$ over all bonds between heavy atoms i and j where $i < j$.
chi1v_C	Carbon valence connectivity index (order 1). This is calculated as the sum of $1/\mathrm{sqrt}(v_i v_j)$ over all bonds between carbon atoms i and j where $i < j$.

density (MOE)	Molecular mass density: Weight divided by vdw_vol (amu/$Å^3$).
Density (Percepta)	ACD/Percepta calculates the density from the molecular weight and the calculated molar volume.
diameter	Largest value in the distance matrix [Petitjean 1992].
h_ema	Sum of hydrogen bond acceptor strengths [Gerber 1998].
h_emd	Sum of hydrogen bond donor strengths [Gerber 1998].
h_emd_C	Sum of hydrogen bond donor strengths of carbon atoms.
h_log_dbo	Sum of log (1 + d-hybrid bond order) for all bonds.
h_log_pbo	Sum of log (1 + pi bond order) for all bonds.
Halogen Ratio	Halogen Ratio
Hetero Ratio	Hetero Ratio
Index of Refraction	ACD/Percepta calculates the refractive index from the molar volume and molar refractivity, according to the Lorentz-Lorenz equation.
Kier1	First kappa shape index: $(n-1)^2 / m^2$ [Hall 1991].
Kier2	Second kappa shape index: $(n-1)^2 / m^2$ [Hall 1991].
Kier3	Third kappa shape index: $(n-1)(n-3)^2 / p_3^2$ for odd n, and $(n-3)(n-2)^2 / p_3^2$ for even n [Hall 1991].
KierFlex	Kier molecular flexibility index: (KierA1) (KierA2) / n [Hall 1991]. (KierA1: First alpha modified shape index: $s(s-1)^2 / m^2$ where s = n + a [Hall 1991].; KierA2: Second alpha modified shape index: $s(s-1)^2 / m^2$ where s = n + a [Hall 1991].)
lip_acc	The number of O and N atoms
lip_don	The number of OH and NH atoms.
Log(BCF)	Bioconcentration Factor (BCF) is used to describe the accumulation of chemicals in organisms, primarily aquatic, that live in contaminated environments.
Log(Koc)	The Adsorption Coefficient (Koc) may be defined as the ratio of the amount of chemical adsorbed per unit weight of organic carbon (oc) in the soil or sediment to the concentration of the chemical in solution at equilibrium.
LogL16 (L)	Partitioning coefficient between gaseous phase and hexadecane
logP(o/w)	Log of the octanol/water partition coefficient (including implicit hydrogens). This property is calculated from a ~100 parameter linear atom type model [LOGP 1998] with r2 = 0.931, RMSE=0.393 on 1,827 molecules.
logS	Log of the aqueous solubility (mol/L). This property is calculated from an atom contribution linear atom type model [Hou 2004] with $r^2 = 0.90$, ~1,200 molecules.
LogS0|LogS0	Intrinsic solubility or solubility for neutral form of compound.
LogS0|RI	Reliability index for LogS0.
LogSw|LogSw	Quantitative solubility in pure water (logSw)
LogSw|pH	Water solubility as a function of pH
McGowan Volume (V)	McGowan characteristic volume - Vx (V)
mr	Molecular refractivity (including implicit hydrogens). This property is calculated from an 11 descriptor linear model [MREF 1998] with $r^2 = 0.997$, RMSE = 0.168 on 1,947 small molecules.
N Ratio	N Ratio
NO Ratio	NO Ratio
Number of Aromatic Rings	Number of Aromatic Rings
Number of Rings (size 3)	Number of rings of size 3
Number of Rings (size 4)	Number of rings of size 4
Number of Rings (size 5)	Number of rings of size 5
Number of Rings (size 6)	Number of rings of size 6
opr_nrot	The number of rotatable bonds from [Oprea 2000].
Parachor	ACD/Percepta calculates the parachor from additive increments. The additive atomic increments were obtained using a database of density, surface tension, and calculated molecular weight.
PEOE_PC-	Total negative partial charge: the sum of the negative q_i.
PEOE_PC+	Total positive partial charge: the sum of the positive q_i.
PEOE_RPC-	Relative negative partial charge: the smallest negative q_i divided by the sum of the negative q_i.
PEOE_RPC+	Relative positive partial charge: the largest positive q_i divided by the sum of the positive q_i.
Pi2 (S)	Polarity / polarizability
Q_PC-	Total negative partial charge: the sum of the negative q_i.
Q_PC+	Total positive partial charge: the sum of the positive q_i.
Q_RPC-	Relative negative partial charge: the smallest negative q_i divided by the sum of the negative q_i.
Q_RPC+	Relative positive partial charge: the largest positive q_i divided by the sum of the positive q_i.
Q_VSA_FHYD	Fractional hydrophobic van der Waals surface area. This is the sum of the v_i such that $\|q_i\|$ is less than or equal to 0.2 divided by the total surface area. The v_i are calculated using a connection table approximation.
Q_VSA_FNEG	Fractional negative van der Waals surface area. This is the sum of the v_i such that q_i is negative divided by the total surface area. The v_i are calculated using a connection table approximation.
Q_VSA_FPNEG	Fractional negative polar van der Waals surface area. This is the sum of the v_i such that q_i is less than -0.2 divided by the total surface area. The v_i are calculated using a connection table approximation.

Q_VSA_FPOL	Fractional polar van der Waals surface area. This is the sum of the v_i such that $\|q_i\|$ is greater than 0.2 divided by the total surface area. The v_i are calculated using a connection table approximation.
Q_VSA_FPOS	Fractional positive van der Waals surface area. This is the sum of the v_i such that q_i is non negative divided by the total surface area. The v_i are calculated using a connection table approximation.
Q_VSA_FPPOS	Fractional positive polar van der Waals surface area. This is the sum of the v_i such that q_i is greater than 0.2 divided by the total surface area. The v_i are calculated using a connection table approximation.
Q_VSA_HYD	Total hydrophobic van der Waals surface area. This is the sum of the v_i such that $\|q_i\|$ is less than or equal to 0.2. The v_i are calculated using a connection table approximation.
Q_VSA_NEG	Total negative van der Waals surface area. This is the sum of the vi such that q_i is negative. The v_i are calculated using a connection table approximation.
Q_VSA_PNEG	Total negative polar van der Waals surface area. This is the sum of the v_i such that q_i is less than -0.2. The v_i are calculated using a connection table approximation.
Q_VSA_POL	Total polar van der Waals surface area. This is the sum of the v_i such that $\|q_i\|$ is greater than 0.2. The v_i are calculated using a connection table approximation.
Q_VSA_POS	Total positive van der Waals surface area. This is the sum of the v_i such that q_i is non-negative. The v_i are calculated using a connection table approximation.
Q_VSA_PPOS	Total positive polar van der Waals surface area. This is the sum of the vi such that q_i is greater than 0.2. The v_i are calculated using a connection table approximation.
R2 (E)	Excessive molar refraction
radius	If r_i is the largest matrix entry in row i of the distance matrix D, then the radius is defined as the smallest of the r_i [Petitjean 1992].
rings	The number of rings.
RPC-	Relative negative partial charge: the smallest negative q_i divided by the sum of the negative q_i.
RPC+	Relative positive partial charge: the largest positive q_i divided by the sum of the positive q_i.
SlogP	Log of the octanol/water partition coefficient. This property is an atomic contribution model [Crippen 1999] that calculates logP from the given structure; i.e. the correct protonation state (washed structures). The training set for SlogP was ~7000 structures.
SMR	Molecular refractivity (including implicit hydrogens). This property is an atomic contribution model [Crippen 1999] that assumes the correct protonation state (washed structures). The model was trained on ~7000 structures.
Surface Tension	ACD/Percepta calculates the surface tension from calculated Molar Volume and calculated Parachor.
TPSA	Polar surface area (Å²) calculated using group contributions to approximate the polar surface area from connection table information only. The parameterization is that of Ertl et al. [Ertl 2000].
VAdjEq	Vertex adjacency information (equality): $-(1-f) \log_2(1-f) - f \log_2 f$ where $f = (n^2 - m) / n^2$, n is the number of heavy atoms and m is the number of heavy-heavy bonds. If f is not in the open interval (0,1), then 0 is returned.
VAdjMa	Vertex adjacency information (magnitude): $1 + \log_2 m$ where m is the number of heavy-heavy bonds. If m is zero, then zero is returned.
VDistEq	If m is the sum of the distance matrix entries then VdistEq is defined to be the sum of $\log_2 m - pi \log_2 pi / m$ where pi is the number of distance matrix entries equal to i.
VDistMa	If m is the sum of the distance matrix entries then VDistMa is defined to be the sum of $\log_2 m - D_{ij} \log_2 D_{ij} / m$ over all i and j.
vdw_area	Area of van der Waals surface (Å²) calculated using a connection table approximation.
vdw_vol	van der Waals volume (Å³) calculated using a connection table approximation.
VP (Temperature (°C) = 25,00)	Vapor pressure
vsa_acc	Approximation to the sum of VDW surface areas (Å²) of pure hydrogen bond acceptors (not counting atoms that are both hydrogen bond donors and acceptors such as -OH).
vsa_don	Approximation to the sum of VDW surface areas of pure hydrogen bond donors (not counting atoms that are both hydrogen bond donors and acceptors such as -OH) (Å²).
vsa_hyd	Approximation to the sum of VDW surface areas of hydrophobic atoms (Å²).
vsa_other	Approximation to the sum of VDW surface areas (Å²) of atoms typed as "other".
vsa_pol	Approximation to the sum of VDW surface areas (Å²) of polar atoms (atoms that are both hydrogen bond donors and acceptors), such as -OH.
Weight	Molecular weight (including implicit hydrogens) in atomic mass units with atomic weights taken from [CRC 1994].
weinerPath	Wiener path number: half the sum of all the distance matrix entries as defined in [Balaban 1979] and [Wiener 1947].
weinerPol	Wiener polarity number: half the sum of all the distance matrix entries with a value of 3 as defined in [Balaban 1979].
zagreb	Zagreb index: the sum of d_i 2 over all heavy atoms i.

*PEOE: The Partial Equalization of Orbital Electronegativities (PEOE) method of calculating atomic partial charges [Gasteiger 1980] is a method in which charge is transferred between bonded atoms until equilibrium.
** Q: Descriptors prefixed with Q_ use the partial charges stored with each structure in the database. In other words, no partial charge calculation is made and it is assumed that some external program has been used to calculate the atomic partial charges.

[CRC 1994] CRC Handbook of Chemistry and Physics. CRC Press (1994).
[Balaban 1982] Balaban, A.T.; Highly Discriminating Distance-Based Topological Index; Chemical Physics Letters 89 No. 5 (1982) 399–404.
[Hall 1991] Hall, L.H., Kier, L.B.; The Molecular Connectivity Chi Indices and Kappa Shape Indices in Structure-Property Modeling; Reviews of Computational Chemistry 2 (1991).
[Hall 1977] Hall, L.H., Kier, L.B.; The Nature of Structure-Activity Relationships and Their Relation to Molecular Connectivity; Eur. J. Med. Chem 12 (1977) 307.
[Petitjean 1992] Petitjean, M.; Applications of the Radius-Diameter Diagram to the Classification of Topological and Geometrical Shapes of Chemical Compounds; J. Chem. Inf. Comput. Sci. 32 (1992) 331–337.
[Gerber 1998] Gerber, P.R.; Charge distribution from a simple molecular orbital type calculation and non-bonding interaction terms in the force field MAB; J. Comput.-Aid. Mol. Des. 12 (1998) 37–51.
[MREF 1998] Labute, P.; MOE Molar Refractivity Model unpublished. Source code in $MOE/svl/quasar.svl/q_mref.svl (1998).
[Oprea 2000] Oprea, Tudor I.; Property Distribution of Drug-Related Chemical Databases; J. Comp. Aid. Mol. Des. 14 (2000) 251–264.
[LOGP 1998] Labute, P.; MOE LogP(Octanol/Water) Model unpublished. Source code in $MOE/svl/quasar.svl/q_logp.svl (1998).
[Hou 2004] Hou, T.J., Xia, K., Zhang, W., Xu, X.J.; ADME Evaluation in Drug Discovery. 4. Prediction of Aqueous Solubility Based on Atom Contribution Approach; J. Chem. Inf. Comput. Sci. 44 (2004) 266–275.
[Crippen 1999] Wildman, S.A., Crippen, G.M.; Prediction of Physiochemical Parameters by Atomic Contributions; J. Chem. Inf. Comput. Sci. 39 No. 5 (1999) 868–873.
[Ertl 2000] Ertl, P., Rohde, B., Selzer, P.; Fast Calculation of Molecular Polar Surface Area as a Sum of Fragment-Based Contributions and Its Application to the Prediction of Drug Transport Properties; J. Med. Chem. 43 (2000) 3714–3717.
[Wiener 1947] Wiener, H.; Structural Determination of Paraffin Boiling Points; Journal of the American Chemical Society 69 (1947) 17–20.
[Balaban 1979] Balaban, A.T.; Five New Topological Indices for the Branching of Tree-Like Graphs; Theoretica Chimica Acta 53 (1979) 355–375.

Table S2 Variable importance (VIP) and uncertainty (95%-confidence level) for the final $^{1}t_{R}$ model

Var ID (Primary)*	VIP	Uncertainty
BP (Pressure (mmHg) = 760,00)	1.645	0.066
LogL16 (L)	1.564	0.030
VAdjMa	1.503	0.072
RT/RI1: Ln(SMR)	1.483	0.069
zagreb	1.482	0.044
VDistMa	1.481	0.069
b_heavy	1.478	0.028
RT/RI1: Ln(a_heavy)	1.476	0.064
RT/RI1: Ln(chi1)	1.469	0.067
RT/RI1: Ln(weight)	1.466	0.044
RT/RI1: Ln(mr)	1.457	0.068
RT/RI1: Ln(chi0)	1.451	0.061
SMR	1.441	0.055
a_heavy	1.435	0.024
weinerPol	1.428	0.094
chi1	1.423	0.024
VAdjEq	1.420	0.067
RT/RI1: Ln(weinerPath)	1.416	0.054
mr	1.415	0.047
Weight	1.412	0.070
chi0	1.411	0.026
chi0v	1.385	0.047
RT/RI1: Ln(Parachor)	1.371	0.060
RT/RI1: Ln(vdw_vol)	1.366	0.058
RT/RI1: Ln(McGowan Volume (V))	1.363	0.062
R2 (E)	1.362	0.058
RT/RI1: Ln(chi1v)	1.360	0.071
RT/RI1: Ln(Kier1)	1.340	0.060
RT/RI1: Ln(vdw_area)	1.321	0.047
Parachor	1.315	0.048
vdw_vol	1.312	0.052
Surface Tension	1.308	0.112
McGowan Volume (V)	1.308	0.051
chi1v	1.301	0.067
RT/RI1: Ln(apol)	1.300	0.073
Kier1	1.284	0.035
Pi2 (S)	1.283	0.140
vdw_area	1.282	0.035
a_hyd	1.270	0.081
Index of Refraction	1.258	0.126
apol	1.245	0.068
vsa_hyd	1.192	0.077
a_nC	1.176	0.076
RT/RI1: Ln (PEOE_PC+)	1.163	0.106
Q_VSA_HYD	1.160	0.090
Q_VSA_NEG	1.157	0.138
RT/RI1: Ln(diameter)	1.151	0.069
VDistEq	1.142	0.053

Var ID (Primary)*	VIP	Uncertainty
RT/RI1: Ln(radius)	1.139	0.066
RT/RI1: Ln(b_count)	1.127	0.072
chi0_C	1.114	0.078
h_log_pbo	1.108	0.111
b_count	1.105	0.085
weinerPath	1.101	0.178
rings	1.095	0.066
Q_PC-	1.094	0.092
Q_PC+	1.094	0.092
chi0v_C	1.082	0.083
a_count	1.074	0.088
PEOE_PC+	1.059	0.173
PEOE_PC-	1.059	0.173
diameter	1.041	0.093
RT/RI1: Ln(Kier2)	1.031	0.056
radius	1.030	0.096
logS	1.027	0.136
Number of Rings (size 6)	0.997	0.137
LogS0|LogS0	0.994	0.135
LogSw|LogSw	0.990	0.139
Density (Percepta)	0.990	0.084
a_aro	0.974	0.093
b_ar	0.973	0.099
Number of Aromatic Rings	0.968	0.118
bpol	0.964	0.096
chi1_C	0.956	0.114
b_single	0.955	0.101
Kier2	0.948	0.115
SlogP	0.929	0.150
Q_RPC-	0.921	0.231
RPC-	0.921	0.231
chi1v_C	0.921	0.130
Q_VSA_POS	0.919	0.186
b_rotN	0.907	0.123
a_nH	0.903	0.110
opr_nrot	0.893	0.133
b_1rotN	0.885	0.136
Kier3	0.825	0.140
h_emd_C	0.798	0.230
b_rotR	0.796	0.075
b_1rotR	0.785	0.083
b_max1len	0.773	0.166
h_emd	0.760	0.233
BetaO (Bo)	0.731	0.217
BetaH2 (B)	0.727	0.219
PEOE_RPC-	0.724	0.159
h_ema	0.715	0.238
density (MOE)	0.713	0.091
balabanJ	0.699	0.087
a_nCl	0.681	0.170
LogS0|RI	0.676	0.147
VP (Temperature (°C) = 25,00)	0.676	0.383

Var ID (Primary)*	VIP	Uncertainty
Log(Koc)	0.673	0.163
lip_acc	0.673	0.258
Log(BCF)	0.673	0.163
logP(o/w)	0.662	0.168
a_nO	0.650	0.286
Q_VSA_FNEG	0.637	0.139
Q_VSA_FPOS	0.637	0.139
a_nF	0.623	0.171
TPSA	0.618	0.194
KierFlex	0.618	0.191
a_acc	0.611	0.307
vsa_acc	0.611	0.207
a_donacc	0.590	0.291
vsa_pol	0.570	0.189
Q_VSA_PPOS	0.566	0.173
PEOE_RPC+	0.551	0.265
b_double	0.551	0.238
C Ratio	0.540	0.098
logP(OW)/weight	0.534	0.133
lip_don	0.533	0.235
vsa_don	0.527	0.290
Halogen Ratio	0.518	0.114
Q_VSA_POL	0.511	0.116
vsa_other	0.505	0.150
Q_VSA_FPNEG	0.501	0.148
b_triple	0.460	0.179
Q_VSA_PNEG	0.458	0.099
Number of Rings (size 5)	0.445	0.172
a_don	0.444	0.214
Q_VSA_FHYD	0.440	0.123
Q_VSA_FPOL	0.440	0.123
Number of Rings (size 3)	0.438	0.380
AlphaH2 (A)	0.423	0.248
a_nN	0.398	0.156
Q_RPC+	0.392	0.196
RPC+	0.392	0.196
a_nS	0.384	0.085
Hetero Ratio	0.367	0.185
N Ratio	0.365	0.167
a_nBr	0.365	0.341
NO Ratio	0.339	0.206
Q_VSA_FPPOS	0.310	0.181
h_log_dbo	0.252	0.128
LogSw|pH	0.230	0.153
a_nP	0.226	0.084
Number of Rings (size 4)	0.172	0.415
a_nI	0.113	0.270

* RT/RI1 is the retention time or retention index model for the first dimension

Table S3 Variable importance (VIP) and uncertainty (95%-confidence level) for the final LRI model

Var ID (Primary)*	VIP	Uncertainty
BP (Pressure (mmHg) = 760,00)	1.636	0.094
LogL16 (L)	1.584	0.031
zagreb	1.493	0.062
b_heavy	1.492	0.040
VAdjMa	1.468	0.071
RT/RI1: Ln(SMR)	1.459	0.073
SMR	1.458	0.060
VDistMa	1.450	0.068
weinerPol	1.447	0.119
RT/RI1: Ln(a_heavy)	1.445	0.062
RT/RI1: Ln(weight)	1.444	0.057
a_heavy	1.443	0.030
Weight	1.439	0.088
RT/RI1: Ln(chi1)	1.438	0.063
RT/RI1: Ln(mr)	1.434	0.071
chi1	1.434	0.031
mr	1.426	0.050
RT/RI1: Ln(chi0)	1.422	0.061
chi0	1.412	0.029
chi0v	1.397	0.050
RT/RI1: Ln(weinerPath)	1.388	0.048
R2 (E)	1.387	0.087
VAdjEq	1.384	0.066
RT/RI1: Ln(Parachor)	1.352	0.060
RT/RI1: Ln(vdw_vol)	1.352	0.057
RT/RI1: Ln(McGowan Volume (V))	1.345	0.061
RT/RI1: Ln(chi1v)	1.333	0.075
vdw_vol	1.324	0.056
Parachor	1.323	0.054
McGowan Volume (V)	1.315	0.056
RT/RI1: Ln(Kier1)	1.315	0.055
chi1v	1.311	0.071
RT/RI1: Ln(vdw_area)	1.303	0.044
a_hyd	1.298	0.093
Pi2 (S)	1.294	0.174
RT/RI1: Ln(apol)	1.287	0.071
vdw_area	1.284	0.038
Kier1	1.282	0.040
apol	1.253	0.074
Surface Tension	1.247	0.119
Index of Refraction	1.236	0.147
vsa_hyd	1.213	0.086
a_nC	1.194	0.083
Q_VSA_HYD	1.190	0.107
Q_VSA_NEG	1.159	0.178

Var ID (Primary)*	VIP	Uncertainty
rings	1.144	0.108
RT/RI1: Ln(diameter)	1.129	0.057
weinerPath	1.128	0.194
chi0_C	1.123	0.081
VDistEq	1.122	0.051
RT/RI1: Ln(radius)	1.122	0.060
RT/RI1: Ln(b_count)	1.121	0.075
RT/RI1: Ln (PEOE_PC+)	1.120	0.110
h_log_pbo	1.118	0.132
b_count	1.108	0.093
chi0v_C	1.088	0.086
Q_PC-	1.074	0.098
Q_PC+	1.074	0.098
a_count	1.074	0.095
logS	1.055	0.164
PEOE_PC+	1.055	0.195
PEOE_PC-	1.055	0.195
Number of Rings (size 6)	1.050	0.174
diameter	1.046	0.110
radius	1.038	0.114
LogS0|LogS0	1.027	0.155
LogSw|LogSw	1.022	0.160
RT/RI1: Ln(Kier2)	1.020	0.054
Density (Percepta)	1.012	0.093
a_aro	0.997	0.102
b_ar	0.997	0.105
Number of Aromatic Rings	0.994	0.125
chi1_C	0.975	0.118
bpol	0.959	0.099
SlogP	0.955	0.181
b_single	0.952	0.104
Kier2	0.950	0.125
chi1v_C	0.933	0.126
Q_RPC-	0.929	0.236
RPC-	0.929	0.236
Q_VSA_POS	0.920	0.189
b_rotN	0.904	0.121
a_nH	0.900	0.105
opr_nrot	0.889	0.129
b_1rotN	0.882	0.132
Kier3	0.821	0.142
b_rotR	0.807	0.062
b_1rotR	0.791	0.068
h_emd_C	0.785	0.258
balabanJ	0.776	0.119
b_max1len	0.765	0.152
h_emd	0.758	0.273
density (MOE)	0.754	0.115
PEOE_RPC-	0.731	0.172

Var ID (Primary)*	VIP	Uncertainty
BetaO (Bo)	0.721	0.255
BetaH2 (B)	0.718	0.257
h_ema	0.692	0.286
Log(Koc)	0.691	0.164
Log(BCF)	0.691	0.164
logP(o/w)	0.686	0.174
a_nCl	0.681	0.242
KierFlex	0.670	0.165
LogS0|RI	0.665	0.107
lip_acc	0.650	0.304
Q_VSA_FNEG	0.650	0.100
Q_VSA_FPOS	0.650	0.100
a_nO	0.644	0.325
a_nF	0.642	0.139
a_acc	0.617	0.341
a_donacc	0.605	0.326
VP (Temperature (°C) = 25,00)	0.603	0.392
vsa_acc	0.595	0.220
Halogen Ratio	0.590	0.135
b_double	0.589	0.309
TPSA	0.580	0.243
PEOE_RPC+	0.578	0.242
vsa_pol	0.549	0.229
Q_VSA_PPOS	0.532	0.142
Q_VSA_FPNEG	0.524	0.100
Q_VSA_POL	0.521	0.105
C Ratio	0.514	0.126
logP(OW)/weight	0.509	0.154
lip_don	0.506	0.215
Q_VSA_FHYD	0.483	0.099
Q_VSA_FPOL	0.483	0.099
vsa_other	0.482	0.130
Q_VSA_PNEG	0.480	0.101
a_nBr	0.457	0.381
a_don	0.450	0.205
Number of Rings (size 5)	0.449	0.165
Number of Rings (size 3)	0.446	0.342
vsa_don	0.429	0.262
b_triple	0.429	0.144
AlphaH2 (A)	0.422	0.241
Q_RPC+	0.420	0.183
RPC+	0.420	0.183
a_nS	0.350	0.116
a_nN	0.345	0.126
Hetero Ratio	0.315	0.219
NO Ratio	0.311	0.254
N Ratio	0.282	0.110
Q_VSA_FPPOS	0.264	0.119
h_log_dbo	0.249	0.099

Var ID (Primary)*	VIP	Uncertainty
LogSw|pH	0.246	0.149
a_nP	0.231	0.072
Number of Rings (size 4)	0.147	0.349
a_nI	0.098	0.231

* RT/RI1 is the retention time or retention index model for the first dimension

Table S4 Variable importance and uncertainty (95%-confidence level) for the final ^2t_R model

Var ID (Primary)*	VIP	Uncertainty
R2 (E)	2.245	0.228
Surface Tension	2.225	0.145
Index of Refraction	2.128	0.271
Pi2 (S)	2.125	0.146
R2 (E)/weight	1.875	0.229
rings	1.826	0.128
vsa_hyd/weight	1.810	0.086
h_log_pbo	1.808	0.173
Number of Rings (size 6)	1.786	0.239
BP (Pressure (mmHg) = 760,00)	1.720	0.203
Number of Aromatic Rings	1.651	0.329
b_ar	1.647	0.279
a_nH/weight	1.636	0.133
a_aro	1.633	0.245
Log(Koc)/weight	1.627	0.086
b_max1len/weight	1.603	0.120
b_single/weight	1.601	0.167
weinerPol/weight	1.587	0.144
vdw_area/weight	1.566	0.125
Pi2 (S)/weight	1.545	0.277
b_rotR/weight	1.536	0.143
Kier3/weight	1.532	0.102
b_1rotR/weight	1.522	0.146
weinerPol	1.495	0.167
balabanJ/weight	1.476	0.132
rings/weight	1.457	0.242
Density (Percepta)	1.452	0.149
Log(BCF)/weight	1.449	0.176
Parachor/weight	1.443	0.100
h_log_pbo/weight	1.437	0.206
McGowan Volume (V)/weight	1.437	0.104
a_count/weight	1.436	0.122
chi0v/weight	1.425	0.108
Kier2/weight	1.401	0.121
bpol/weight	1.400	0.131
radius/weight	1.362	0.103
VDistEq/weight	1.355	0.169

Var ID (Primary)*	VIP	Uncertainty
zagreb	1.330	0.166
apol/weight	1.325	0.089
diameter/weight	1.318	0.109
opr_nrot/weight	1.317	0.154
b_count/weight	1.316	0.117
LogL16 (L)	1.314	0.195
b_1rotR	1.309	0.136
b_rotR	1.304	0.128
Number of Rings (size 6)/weight	1.302	0.250
Number of Aromatic Rings/weight	1.302	0.285
RT/RI2: Ln(blabanJ)	1.296	0.166
KierFlex/weight	1.292	0.307
b_ar/weight	1.282	0.230
b_1rotN/weight	1.282	0.157
b_rotN/weight	1.267	0.146
a_aro/weight	1.264	0.196
RT/RI2: -1.5root (b_max1len)	1.264	0.159
logP(OW)/weight	1.254	0.095
logP(o/w)/weight	1.254	0.095
vdw_vol/weight	1.251	0.086
chi1v_C/weight	1.248	0.079
chi0v_C/weight	1.238	0.074
SlogP/weight	1.198	0.201
balabanJ	1.197	0.169
C Ratio/weight	1.193	0.111
VAdjMa	1.185	0.202
chi1v/weight	1.167	0.119
BetaO (Bo)	1.158	0.224
Kier1/weight	1.155	0.183
BetaH2 (B)	1.152	0.229
LogL16 (L)/weight	1.136	0.271
zagreb/weight	1.131	0.091
b_heavy	1.104	0.162
b_max1len	1.099	0.206
LogSw|pH/weight	1.096	0.191
Q_VSA_HYD/weight	1.093	0.196
vsa_pol	1.069	0.366
TPSA	1.061	0.187
chi0_C/weight	1.057	0.055
VAdjEq/weight	1.046	0.170
VDistMa	1.044	0.205
Q_VSA_FPOS/weight	1.043	0.213
mr/weight	1.037	0.093
LogS0|RI/weight	1.032	0.178
h_emd	1.030	0.176
h_ema	1.030	0.150
Kier3	1.030	0.184
Weight	1.027	0.242

Var ID (Primary)*	VIP	Uncertainty
a_nH	1.021	0.191
SMR/weight	1.016	0.094
C Ratio	1.014	0.049
BetaO (Bo)/weight	1.011	0.271
Q_VSA_FPNEG/weight	1.008	0.339
Q_VSA_NEG/weight	1.001	0.306
BetaH2 (B)/weight	1.001	0.272
Q_VSA_PPOS	0.998	0.273
density (MOE)	0.996	0.137
KierFlex	0.984	0.226
lip_acc	0.980	0.165
vsa_don	0.979	0.574
VDistMa/weight	0.977	0.145
Q_VSA_FHYD/weight	0.974	0.149
Q_VSA_PNEG/weight	0.967	0.230
vsa_don/weight	0.963	0.502
a_nN	0.956	0.208
lip_don	0.954	0.300
VAdjMa/weight	0.954	0.142
a_hyd/weight	0.942	0.081
Q_VSA_FPPOS	0.940	0.319
chi1_C/weight	0.939	0.075
Hetero Ratio	0.920	0.193
opr_nrot	0.920	0.189
b_1rotN	0.914	0.180
a_nC/weight	0.909	0.060
a_donacc	0.905	0.313
a_nN/weight	0.894	0.278
a_nS	0.893	0.181
N Ratio	0.893	0.257
a_nS/weight	0.890	0.231
a_nF	0.889	0.268
b_rotN	0.889	0.165
TPSA/weight	0.884	0.235
NO Ratio	0.881	0.203
Q_VSA_POS/weight	0.879	0.114
vsa_other	0.877	0.210
Index of Refraction/weight	0.876	0.128
b_single	0.875	0.208
a_heavy	0.874	0.164
h_ema/weight	0.868	0.168
logS/weight	0.863	0.137
Q_VSA_FNEG/weight	0.862	0.118
a_nF/weight	0.853	0.228
vsa_pol/weight	0.850	0.350
chi1	0.846	0.158
Log(Koc)	0.846	0.164
Log(BCF)	0.846	0.164
lip_acc/weight	0.843	0.215

Var ID (Primary)*	VIP	Uncertainty
b_heavy/weight	0.836	0.097
Q_VSA_FPNEG	0.831	0.188
b_double/weight	0.827	0.819
chi0/weight	0.821	0.193
a_don	0.814	0.237
Q_VSA_PPOS/weight	0.814	0.325
b_double	0.806	0.760
N Ratio/weight	0.805	0.296
VAdjEq	0.804	0.185
Q_VSA_FPOL/weight	0.802	0.292
vsa_acc	0.800	0.343
chi0	0.798	0.161
h_emd/weight	0.792	0.227
PEOE_PC+	0.790	0.133
PEOE_PC-	0.790	0.133
lip_don/weight	0.786	0.284
a_acc	0.779	0.299
Q_VSA_POL/weight	0.770	0.200
BP (Pressure (mmHg) = 760,00)/weight	0.763	0.093
Kier2	0.756	0.174
LogSw|LogSw/weight	0.753	0.143
vsa_other/weight	0.753	0.186
Q_VSA_PNEG	0.752	0.272
AlphaH2 (A)	0.752	0.272
LogS0|LogS0/weight	0.751	0.140
SMR	0.748	0.177
Q_VSA_POL	0.741	0.243
a_donacc/weight	0.730	0.299
VP (Temperature (°C) = 25,00)	0.727	0.475
Q_VSA_FHYD	0.722	0.140
Q_VSA_FPOL	0.722	0.140
chi1/weight	0.722	0.036
density (MOE)/weight	0.721	0.199
h_emd_C	0.714	0.115
mr	0.702	0.166
a_heavy/weight	0.696	0.072
Q_RPC-/weight	0.690	0.187
RPC-/weight	0.690	0.187
bpol	0.683	0.150
vsa_acc/weight	0.679	0.411
VP (Temperature (°C) = 25,00)/weight	0.676	0.433
PEOE_RPC+/weight	0.675	0.319
chi1v_C	0.665	0.149
Halogen Ratio	0.663	0.078
Surface Tension/weight	0.659	0.218
chi0v	0.656	0.163
a_nO	0.656	0.175

Var ID (Primary)*	VIP	Uncertainty
Q_VSA_NEG	0.653	0.137
Hetero Ratio/weight	0.647	0.228
a_nCl	0.647	0.127
chi1v	0.640	0.157
a_don/weight	0.639	0.234
Q_VSA_FPPOS/weight	0.638	0.318
Q_PC+	0.637	0.130
Q_PC-	0.637	0.130
logP(o/w)	0.632	0.152
PEOE_RPC-/weight	0.629	0.237
a_acc/weight	0.626	0.296
Halogen Ratio/weight	0.625	0.106
Number of Rings (size 5)	0.622	0.207
Number of Rings (size 5)/weight	0.613	0.227
a_nBr	0.603	0.766
a_nCl/weight	0.593	0.143
h_log_dbo	0.593	0.149
a_count	0.573	0.145
NO Ratio/weight	0.569	0.229
PEOE_RPC+	0.566	0.269
Q_RPC+	0.558	0.149
RPC+	0.558	0.149
diameter	0.557	0.156
h_log_dbo/weight	0.555	0.171
Number of Rings (size 3)	0.554	0.361
b_triple	0.552	0.163
Number of Rings (size 3)/weight	0.543	0.350
radius	0.541	0.164
Q_VSA_HYD	0.536	0.176
a_nC	0.530	0.102
a_hyd	0.529	0.154
chi1_C	0.517	0.080
a_nP	0.517	0.155
Q_VSA_FNEG	0.516	0.178
Q_VSA_FPOS	0.516	0.178
chi0v_C	0.506	0.094
Q_PC-/weight	0.506	0.153
Q_PC+/weight	0.506	0.153
b_count	0.506	0.119
a_nO/weight	0.495	0.142
vdw_vol	0.493	0.109
Kier1	0.492	0.090
PEOE_PC+/weight	0.487	0.197
PEOE_PC-/weight	0.487	0.197
Parachor	0.485	0.096
a_nP/weight	0.477	0.192
vdw_area	0.474	0.072
b_triple/weight	0.474	0.142
AlphaH2 (A)/weight	0.474	0.155

Var ID (Primary)*	VIP	Uncertainty
Density (Percepta)/weight	0.471	0.114
McGowan Volume (V)	0.470	0.083
chi0_C	0.468	0.083
VDistEq	0.468	0.068
apol	0.452	0.063
Q_RPC+/weight	0.450	0.202
RPC+/weight	0.450	0.202
SlogP	0.447	0.108
vsa_hyd	0.447	0.083
h_emd_C/weight	0.432	0.092
logS	0.427	0.135
LogS0|LogS0	0.413	0.179
Q_VSA_POS	0.412	0.158
LogSw|LogSw	0.411	0.178
weinerPath/weight	0.409	0.110
weinerPath	0.401	0.128
a_nBr/weight	0.398	0.233
PEOE_RPC-	0.383	0.115
LogS0|RI	0.360	0.268
Q_RPC-	0.357	0.163
RPC-	0.357	0.163
LogSw|pH	0.343	0.361
Number of Rings (size 4)/weight	0.256	0.582
Number of Rings (size 4)	0.209	0.456
a_nI	0.150	0.358
a_nI/weight	0.150	0.358

* RT/RI2 is the retention time or retention index model for the second dimension

Table S5 Variable importance and uncertainty (95%-confidence level) for the final PEG-^2I model

Var ID (Primary)*	VIP	Uncertainty
Surface Tension	2.151	0.170
R2 (E)	2.140	0.268
Index of Refraction	2.061	0.300
Pi2 (S)	1.974	0.117
R2 (E)/weight	1.887	0.282
vsa_hyd/weight	1.778	0.175
rings	1.728	0.207
Number of Rings (size 6)	1.707	0.337
h_log_pbo	1.704	0.210
a_nH/weight	1.661	0.146
b_single/weight	1.656	0.175
b_max1len/weight	1.609	0.148
Pi2 (S)/weight	1.594	0.291
vdw_area/weight	1.579	0.113
Number of Aromatic Rings	1.576	0.401

Var ID (Primary)*	VIP	Uncertainty
b_ar	1.575	0.355
Kier3/weight	1.572	0.149
a_aro	1.563	0.322
b_rotR/weight	1.535	0.139
Density (Percepta)	1.527	0.152
Log(Koc)/weight	1.525	0.206
b_1rotR/weight	1.523	0.154
KierFlex/weight	1.523	0.315
chi0v/weight	1.499	0.170
a_count/weight	1.495	0.150
BP (Pressure (mmHg) = 760,00)	1.494	0.215
Parachor/weight	1.485	0.138
McGowan Volume (V)/weight	1.482	0.137
weinerPol/weight	1.478	0.226
Kier2/weight	1.473	0.123
bpol/weight	1.466	0.130
Log(BCF)/weight	1.464	0.242
h_log_pbo/weight	1.461	0.324
rings/weight	1.459	0.267
opr_nrot/weight	1.436	0.160
b_1rotR	1.406	0.164
b_rotR	1.402	0.159
b_1rotN/weight	1.400	0.148
b_count/weight	1.394	0.159
b_rotN/weight	1.390	0.139
apol/weight	1.386	0.155
radius/weight	1.376	0.066
diameter/weight	1.369	0.097
RT/RI2: -1.5root (b_max1len)	1.352	0.165
Number of Aromatic Rings/weight	1.332	0.404
b_ar/weight	1.324	0.364
a_aro/weight	1.318	0.339
Number of Rings (size 6)/weight	1.317	0.276
vdw_vol/weight	1.311	0.158
chi0v_C/weight	1.309	0.168
Kier1/weight	1.304	0.156
chi1v_C/weight	1.296	0.121
RT/RI2: Ln(blabanJ)	1.294	0.148
chi1v/weight	1.280	0.146
logP(OW)/weight	1.266	0.159
logP(o/w)/weight	1.266	0.159
weinerPol	1.256	0.174
balabanJ/weight	1.244	0.121
b_max1len	1.227	0.210
balabanJ	1.214	0.150
a_nH	1.208	0.197
SlogP/weight	1.198	0.300
Kier3	1.183	0.187
VDistEq/weight	1.178	0.170
Q_VSA_HYD/weight	1.169	0.177
KierFlex	1.149	0.269
density (MOE)	1.140	0.160
zagreb/weight	1.132	0.223
chi0_C/weight	1.125	0.167
LogL16 (L)/weight	1.103	0.228
opr_nrot	1.094	0.179
LogL16 (L)	1.090	0.192
b_1rotN	1.087	0.166
mr/weight	1.084	0.163
zagreb	1.081	0.167
b_single	1.078	0.221
BetaO (Bo)	1.071	0.244
C Ratio	1.068	0.155
BetaH2 (B)	1.065	0.246
b_rotN	1.065	0.153
SMR/weight	1.039	0.165
a_nS	1.028	0.180
a_nS/weight	1.021	0.253
C Ratio/weight	0.997	0.186
a_hyd/weight	0.996	0.203
chi0/weight	0.993	0.208
vsa_don	0.989	0.554
BetaO (Bo)/weight	0.988	0.290
Q_VSA_NEG/weight	0.987	0.443
vsa_pol	0.985	0.361
Log(Koc)	0.982	0.152
Log(BCF)	0.982	0.153
a_nC/weight	0.979	0.141
BetaH2 (B)/weight	0.978	0.288
vsa_don/weight	0.977	0.493
a_nF	0.975	0.453
chi1_C/weight	0.973	0.126
Q_VSA_POS/weight	0.960	0.107
Kier2	0.959	0.168
h_emd	0.949	0.229
TPSA	0.945	0.154
a_nF/weight	0.945	0.461
lip_don	0.939	0.378
Q_VSA_PPOS	0.927	0.223
a_nN	0.926	0.149
VAdjMa	0.921	0.119
Q_VSA_FPNEG/weight	0.920	0.246
logS/weight	0.913	0.155
Q_VSA_PNEG/weight	0.913	0.245
Q_VSA_FPPOS	0.906	0.215
h_ema	0.896	0.209
VP (Temperature (°C) = 25,00)	0.893	0.356

Var ID (Primary)*	VIP	Uncertainty
bpol	0.888	0.162
chi1v_C	0.882	0.136
b_heavy/weight	0.880	0.185
a_nN/weight	0.876	0.229
Hetero Ratio	0.873	0.227
N Ratio	0.873	0.215
b_heavy	0.865	0.160
lip_acc	0.862	0.134
VP (Temperature (°C) = 25,00)/weight	0.857	0.361
Q_VSA_FPOS/weight	0.856	0.163
a_donacc	0.854	0.378
b_double/weight	0.851	0.800
TPSA/weight	0.842	0.198
a_heavy/weight	0.830	0.120
vsa_pol/weight	0.822	0.345
Q_VSA_FHYD/weight	0.821	0.127
chi1/weight	0.821	0.089
a_count	0.813	0.171
NO Ratio	0.811	0.160
h_ema/weight	0.811	0.190
LogSw|pH/weight	0.808	0.197
Q_RPC+	0.807	0.208
RPC+	0.807	0.208
logP(o/w)	0.805	0.149
b_double	0.803	0.738
Q_VSA_FPNEG	0.803	0.197
N Ratio/weight	0.801	0.307
Q_VSA_PPOS/weight	0.800	0.238
AlphaH2 (A)	0.799	0.457
a_don	0.799	0.258
h_emd/weight	0.793	0.271
VDistMa/weight	0.790	0.168
lip_acc/weight	0.787	0.148
lip_don/weight	0.784	0.346
vsa_other	0.783	0.201
Weight	0.776	0.293
diameter	0.774	0.125
LogS0|RI/weight	0.770	0.200
VDistMa	0.767	0.140
Surface Tension/weight	0.764	0.175
VAdjEq/weight	0.763	0.177
LogSw|LogSw/weight	0.762	0.163
radius	0.761	0.104
Halogen Ratio	0.760	0.129
LogS0|LogS0/weight	0.760	0.161
VAdjMa/weight	0.750	0.165
Q_VSA_POL/weight	0.743	0.205
chi0v_C	0.742	0.136

Var ID (Primary)*	VIP	Uncertainty
Q_VSA_PNEG	0.735	0.275
b_count	0.735	0.162
h_log_dbo	0.734	0.235
a_nP	0.733	0.287
Q_VSA_FPOL/weight	0.729	0.264
a_acc	0.724	0.408
a_nBr	0.723	0.793
a_donacc/weight	0.716	0.370
Q_VSA_FHYD	0.715	0.187
Q_VSA_FPOL	0.715	0.187
BP (Pressure (mmHg) = 760,00)/weight	0.715	0.098
a_nP/weight	0.714	0.293
h_log_dbo/weight	0.707	0.238
vsa_other/weight	0.707	0.229
Halogen Ratio/weight	0.704	0.177
Q_VSA_POL	0.695	0.271
PEOE_RPC+	0.694	0.180
vsa_acc	0.684	0.383
a_nCl	0.680	0.136
a_nCl/weight	0.678	0.143
chi1_C	0.673	0.099
Q_VSA_FPPOS/weight	0.667	0.227
VDistEq	0.667	0.084
Q_VSA_FNEG/weight	0.664	0.192
Hetero Ratio/weight	0.656	0.243
a_heavy	0.649	0.177
chi0_C	0.645	0.109
chi1	0.644	0.158
a_don/weight	0.641	0.278
PEOE_PC+	0.631	0.181
PEOE_PC-	0.631	0.181
vsa_acc/weight	0.631	0.507
Q_VSA_FNEG	0.630	0.177
Q_VSA_FPOS	0.630	0.177
Number of Rings (size 5)/weight	0.629	0.301
h_emd_C	0.621	0.222
PEOE_RPC+/weight	0.620	0.291
Index of Refraction/weight	0.617	0.143
Number of Rings (size 3)	0.612	0.420
Number of Rings (size 5)	0.611	0.266
chi1v	0.608	0.116
Q_VSA_HYD	0.606	0.150
b_triple	0.604	0.201
a_acc/weight	0.603	0.420
a_nC	0.602	0.078
Number of Rings (size 3)/weight	0.600	0.420
Q_PC+/weight	0.587	0.195
Q_PC-/weight	0.587	0.195

Var ID (Primary)*	VIP	Uncertainty
Q_VSA_POS	0.585	0.158
NO Ratio/weight	0.582	0.183
vsa_hyd	0.577	0.126
a_nO	0.575	0.260
chi0	0.574	0.201
weinerPath/weight	0.570	0.127
apol	0.569	0.125
SMR	0.566	0.169
SlogP	0.552	0.160
VAdjEq	0.550	0.152
b_triple/weight	0.548	0.234
density (MOE)/weight	0.539	0.155
mr	0.534	0.166
a_nBr/weight	0.530	0.212
Q_RPC+/weight	0.529	0.203
RPC+/weight	0.529	0.203
Q_PC+	0.507	0.183
Q_PC-	0.507	0.183
PEOE_PC+/weight	0.506	0.138
PEOE_PC-/weight	0.506	0.138
vdw_vol	0.495	0.123
AlphaH2 (A)/weight	0.492	0.163
Density (Percepta)/weight	0.491	0.097
PEOE_RPC-/weight	0.491	0.352
McGowan Volume (V)	0.485	0.118
Q_VSA_NEG	0.482	0.212
a_nO/weight	0.482	0.153
Parachor	0.479	0.121
a_hyd	0.476	0.110
weinerPath	0.475	0.147
Q_RPC-/weight	0.474	0.190
RPC-/weight	0.474	0.190
logS	0.474	0.124
chi0v	0.465	0.183
Kier1	0.454	0.162
vdw_area	0.454	0.109
PEOE_RPC-	0.445	0.089
h_emd_C/weight	0.444	0.198
LogS0|RI	0.440	0.463
LogSw|pH	0.413	0.337
LogS0|LogS0	0.381	0.140
LogSw|LogSw	0.381	0.138
Q_RPC-	0.374	0.110
RPC-	0.374	0.110
Number of Rings (size 4)/weight	0.336	0.499
Number of Rings (size 4)	0.266	0.233
a_nI	0.186	0.439
a_nI/weight	0.186	0.439

* RT/RI2 is the retention time or retention index model for the second dimension

Table S6 Experimental and predicted values for the external validation set using PLS and ChromGenius

Compound	$^{1}t_R$ (s) * experi-mental	$^{1}t_R$ (s) * PLS	$^{1}t_R$ (s) * Chrom Genius	LRI * experi-mental	LRI * PLS	LRI * Chrom Genius	$^{2}t_R$ (s) * experi-mental	$^{2}t_R$ (s) * PLS	$^{2}t_R$ (s) * Chrom Genius	PEG-^{2}I * experi-mental	PEG-^{2}I * PLS	PEG-^{2}I * Chrom Genius
1,1,1,2-Tetrachloroethane	365	310	451	861	823	871	2.16	2.15	2.27			
1,1,2,2-Tetrachloroethane	470	310	432	920	817	894	2.43	2.39	2.22	34.3	59.8	32.7
1,2,3,4,7,8,9-HpCDF	2985	2869	2705	2950	2756	2898	4.44	4.34	4.21	111.4	104.2	101.0
1,2,3,7,8-PeCDF	2585	2571	2456	2484	2496	2552	3.99	4.23	4.32	92.9	99.5	107.5
1,2,4-Trimethylbenzene	595	603	585	991	926	994	2.27	2.06	2.26	25.2	15.6	26.4
1,2-Diethylbenzene	715	726	658	1058	1009	1053	2.32	2.22	2.27	26.5	22.3	23.0
1,3,5-Triethylbenzene	995	1009	1194	1218	1198	1205	2.28	2.24	2.33	22.7	19.6	23.9
1,3-Dichlorobenzene	620	692	514	1006	1004	1004	2.51	2.54	2.60	36.0	34.3	37.2
1,4-Dichlorobenzene	635	702	515	1014	1024	990	2.56	2.60	2.55	38.2	38.3	40.2
1,4-Phenylenediamine	1070	1463	1656	1262	1421	1340	4.30	5.88	3.61	115.8	171.8	92.0
1-bromohexadecane	2055	1929	1808	1974	1900	1954	2.26	2.21	2.20	15.7	11.9	6.7
1-diphenoxyphosphoryloxy-2-tert-butyl-benzene	2695	2826	2745	2606	2729	2586	4.21	4.18	3.96	102.3	106.9	86.4
1-Methyl-2-n-propylbenzene	725	761	658	1064	1030	1066	2.30	2.18	2.29	25.5	19.6	23.9
1-Methyl-3-n-propylbenzene	700	761	658	1050	1031	1055	2.24	2.18	2.27	22.9	19.5	23.1
1-Pentanol	295	153	403	822	796	846	1.77	1.82	1.97			
2 Phenyl-2-propanol	760	910	867	1083	1111	1079	2.74	2.72	2.88	45.6	41.0	50.9
2,4,5-Trichlorophenol	1220	1343	1271	1355	1474	1330	3.08	3.12	3.12	57.8	61.2	47.8
2,4,6-Trichlorophenol	1210	1300	1176	1348	1444	1304	3.09	3.03	3.05	58.4	56.1	55.9
2,4'-DDE	2200	2276	2188	2100	2217	2127	3.73	3.80	3.70	83.4	84.9	81.7
2,4'-DDT	2375	2329	2380	2270	2246	2274	3.88	3.64	3.82	88.7	79.4	86.1
2,6-Dichlorophenol	965	1029	926	1200	1251	1160	3.16	2.88	2.81	63.7	50.2	57.8
2-Acetylaminofluorene	2490	2386	2410	2385	2276	2189	5.26	4.95	4.91	152.0	138.7	137.1
2-Butoxy-2-oxoethyl butyl phthalate	2405	2413	2315	2300	2352	2209	3.44	3.70	3.89	68.1	76.5	69.6
2-Ethyltoluene	570	588	522	977	923	974	2.28	2.13	2.24	26.0	21.9	23.6
2-Phenylphenol	1460	1626	1604	1514	1677	1603	3.58	3.95	3.99	79.6	98.3	99.0
3,5-Dichlorobenzoic acid methyl ester	1265	1349	1261	1384	1442	1428	2.84	3.15	3.41	46.1	60.6	64.3
3,6-Dibromocarbazole	2690	2574	2741	2600	2449	2547	5.62	4.96	5.14	167.6	137.9	169.2
3-methyl-2-(3H)-benzothiazolon	1545	1473	1555	1572	1562	1472	4.41	4.22	4.07	117.8	111.5	125.1
4,4'-DDD	2370	2212	2243	2265	2157	2193	3.91	3.69	3.83	90.2	80.7	86.7
4-Bromodiphenyl ether	1710	1790	1791	1693	1814	1732	3.53	3.72	3.60	76.2	78.7	82.7
4-Chloroaniline	965	1122	819	1200	1248	1181	3.55	3.90	3.14	81.7	91.2	87.2
4-Chlorotoluene	535	575	480	957	934	944	2.37	2.31	2.40	30.6	29.5	32.1
4-Methoxybenzyl alcohol	1105	1095	1072	1282	1240	1316	3.42	3.38	3.33	74.8	67.2	98.1
4-Nitroaniline	1575	1435	1265	1593	1469	1679	4.96	4.65	4.78	143.1	129.2	136.5
7,12-Dimethylbenz(a)anthracene	2840	2585	2634	2771	2530	2650	5.35	4.74	4.91	154.3	130.4	126.0
a,a-Dimethylphenethylamine (phentermine)	905	1189	970	1166	1238	1141	2.63	3.14	2.43	39.5	55.8	34.4
Acequinocyl	2910	3039	2891	2856	2970	2845	3.35	4.08	3.68	61.3	95.9	51.6
Acetochlor	1935	1907	2043	1871	1900	1929	3.31	3.42	3.30	65.0	67.0	59.8
Aclonifen	2370	2448	2294	2265	2261	2741	4.63	4.76	4.23	123.5	127.2	119.3
Aldrin	2045	2319	2144	1965	2233	2019	3.35	3.83	3.63	66.2	80.0	81.0
Aniline	565	823	702	974	1018	992	3.07	3.93	2.78	62.6	95.0	52.0
Anthracene	1835	1814	1653	1788	1856	1742	4.09	4.28	4.06	101.7	109.6	96.0
BDE-154	2985	3023	3243	2950	2982	3023	5.10	4.84	5.31	141.9	134.3	147.0
Benzo(c)phenanthrene	2495	2415	2385	2390	2388	2385	4.98	4.94	4.86	139.0	140.7	128.5
Benzo(g,h,i)perylene	3200	3276	3093	3236	3140	2964	6.41	6.23	6.38	200.5	201.2	197.1

Compound	$^{1}t_R$ (s) *			LRI *			$^{2}t_R$ (s) *			PEG-^{2}I *		
	experi-mental	PLS	Chrom Genius	experi-mental	PLS	Chrom Genius	experi-mental	PLS	Chrom Genius	experi-mental	PLS	Chrom Genius
Benzo(k)fluoranthene	2845	2846	2771	2776	2726	2793	5.32	5.42	5.63	152.9	159.7	169.9
Benzoic acid, benzyl ester	1805	1837	1790	1765	1817	1809	3.70	3.74	3.77	83.7	85.1	80.4
Benzothiazole	1005	1180	912	1224	1325	1206	3.75	3.71	3.52	90.7	79.9	71.0
Bis(2-n-butoxyethyl)phthalate	2560	2602	2440	2458	2525	2553	3.36	3.59	3.53	63.8	69.4	68.8
Bisphenol C	2365	2310	2468	2260	2216	2282	4.48	4.11	4.40	116.6	100.8	130.1
Bisphenol PH	3220	3553	3224	3264	3322	3197	5.58	5.32	5.09	161.9	155.0	144.9
Bisphenol TMC	2745	2723	2793	2661	2578	2643	4.52	4.26	4.64	116.4	106.3	113.5
Butylparaben	1750	1603	1661	1723	1657	1683	3.37	3.53	3.40	68.7	77.3	65.2
Caffeine	1890	1857	1956	1833	1852	1985	4.84	4.84	4.97	136.1	132.6	159.1
Chlorbenside	2190	2191	2082	2091	2128	2245	4.00	3.98	3.63	95.9	91.2	79.8
Chlorobenzilate	2350	2459	2258	2245	2366	2215	3.64	4.12	3.94	77.8	98.9	92.8
Chlorthiophos	2350	2387	2322	2245	2288	2213	3.76	3.74	3.77	83.4	85.9	81.1
Cholesterol	3105	3103	2817	3107	2984	2833	3.25	3.65	2.89	55.1	72.0	46.2
Chrysene	2565	2454	2385	2463	2405	2385	5.09	4.96	5.03	143.9	140.7	140.8
Cinnamyl alcohol	1140	1182	1040	1303	1298	1284	3.34	3.38	3.10	70.8	69.5	64.6
cis-10-Heptadecenoic acid methyl ester (C18)	2090	2098	1961	2004	2000	1960	2.31	2.33	2.30	17.8	19.0	17.4
cis-11,14,17-Eicosatrienoic acid methyl ester (C21)	2405	2388	2356	2300	2282	2224	2.51	2.51	2.50	25.0	25.5	24.0
cis-Nonachlor	2230	2431	2415	2129	2369	2319	3.31	3.79	3.71	63.6	79.2	79.2
Coumaphos	2780	2577	2328	2700	2509	2327	4.50	4.34	3.91	115.3	113.9	91.9
Cypermethrin isomer 1	2870	2878	2883	2806	2750	2787	3.86	3.84	3.92	85.2	86.2	87.3
Cypermethrin isomer 2	2885	2878	2883	2825	2750	2787	3.83	3.84	3.92	83.7	86.2	87.3
delta-BHC	1860	1398	1755	1808	1554	1657	4.13	3.23	3.39	103.4	61.6	72.5
Dibenz(a,j)acridine	3125	3068	3092	3133	2924	3057	5.87	5.63	6.28	176.2	171.2	195.3
Diethyl phthalate	1560	1610	1641	1583	1645	1641	3.41	3.50	3.63	71.4	72.4	83.5
Diflufenican	2495	2614	2617	2390	2509	2425	3.35	4.19	3.83	63.5	112.8	82.6
Diisobutyl phthalate	1915	1932	1914	1854	1916	1831	3.06	3.21	3.17	53.6	56.4	57.0
Diisopentyl phthalate	2150	2135	2114	2057	2100	2054	3.04	3.15	3.10	51.5	52.6	52.8
Diphenamid	2100	2069	2041	2013	2041	2084	4.42	4.01	3.60	115.5	96.7	72.0
Diphenyl ether	1290	1438	1494	1400	1523	1557	3.27	3.38	3.21	65.8	64.1	64.7
d-Limonene	665	751	628	1031	1049	1027	2.05	2.21	2.20	14.4	23.0	15.0
Endosulfan ether	1895	2039	1658	1838	2026	1821	3.64	3.64	3.50	80.5	73.4	70.8
Endosulfan II	2350	2626	2612	2245	2540	2448	4.23	4.68	4.84	105.2	122.9	136.3
Endrin	2325	2321	2268	2220	2227	2244	4.10	3.69	3.99	99.4	74.4	95.5
Endrin ketone	2540	2484	2473	2437	2351	2512	5.12	4.30	4.09	145.3	98.1	90.0
Ethalfluralin	1650	1858	1782	1648	1833	1739	2.32	2.73	2.47	20.5	36.5	27.2
Ethyl methacrylate	290	320	517	819	785	921	1.84	2.10	1.97			
Ethyl methanesulfonate	485	474	783	929	795	1142	3.13	3.27	3.63	66.5	84.3	58.7
Ethylan (Perthane)	2335	2254	2357	2230	2173	2223	3.61	3.39	3.17	76.6	66.1	66.0
Ethylparaben	1470	1366	1236	1521	1483	1579	3.43	3.67	3.71	72.6	85.5	82.1
Fenpropimorph	2070	2227	2233	1987	2171	2082	2.54	3.13	3.75	28.6	49.1	69.4
Fenvalerate	2970	3098	3007	2931	3011	2960	4.09	4.30	4.24	95.3	107.3	105.1
Fipronil	2140	2503	2638	2048	2292	2383	2.83	3.94	2.05	41.8	95.5	43.3
Fluazifop-p-butyl	2340	2387	2387	2235	2340	2209	2.91	3.39	3.46	44.1	68.7	74.4
Fluquinconazole	2780	3137	2718	2700	2941	2491	4.67	5.25	3.92	123.2	148.7	89.0
Flusilazole	2290	2287	2314	2186	2141	2286	3.64	3.61	3.24	78.4	73.3	59.6
Folpet	2170	2198	2192	2074	2138	1912	4.85	4.56	4.46	135.3	124.2	119.4
gamma-BHC (Lindane)	1790	1398	1755	1754	1554	1657	3.79	3.23	3.39	88.0	61.6	72.5

Compound	$^{1}t_R$ (s) *			LRI *			$^{2}t_R$ (s) *			PEG-^{2}I *		
	experi-mental	PLS	Chrom Genius	experi-mental	PLS	Chrom Genius	experi-mental	PLS	Chrom Genius	experi-mental	PLS	Chrom Genius
hentriacontane	3100	3117	3220	3100	3099	3175	2.07	2.07	2.06	0.5	0.3	0.3
Heptachlor	1965	2160	2042	1896	2135	1937	3.26	3.72	3.55	62.5	77.8	77.0
Hexachlorocyclopentadiene	1175	1424	1000	1326	1512	1312	2.59	3.04	2.90	35.6	66.1	92.0
Hexachloropropene	965	1010	718	1200	1194	1138	2.64	2.36	2.64	39.6	41.3	12.9
icosane	2085	2076	1966	2000	2002	1985	1.93	1.93	1.91	0.2	0.2	0.3
Merphos	2155	1955	1941	2061	1930	2146	2.90	2.89	2.97	45.0	47.9	5.3
Methyl 2-nonynoate	1130	984	909	1297	1108	1211	2.60	2.01	2.34	36.6	18.5	18.3
Methyl decanoate (C11)	1170	1226	975	1323	1318	1282	2.17	2.14	2.12	16.3	15.9	17.6
Methyl eugenol	1290	1299	1443	1400	1431	1376	3.01	3.18	3.20	53.7	54.5	52.9
Methyl lignocerate (C25)	2805	2783	2764	2729	2724	2737	2.26	2.27	2.25	11.4	11.7	11.0
Methyl undecanoate (C12)	1325	1378	1066	1423	1419	1362	2.18	2.17	2.15	15.2	16.0	15.5
Musk Ketone	2040	2017	2193	1961	1998	1866	3.58	3.42	3.11	76.9	65.2	57.8
n-Decylbenzene	1685	1678	1461	1674	1717	1660	2.34	2.42	2.34	21.3	24.5	20.7
N-ethyl-p-toluensulfonamide	1730	1632	1599	1708	1613	1611	4.21	4.03	3.86	107.6	105.0	93.6
nonacosane	2945	2954	2970	2900	2899	2864	2.04	2.04	2.03	0.5	0.3	0.3
Octachlorostyrene	2120	2259	1962	2030	2185	2106	3.31	3.57	3.61	64.1	74.5	72.7
octacosane	2865	2868	2880	2800	2799	2764	2.03	2.03	2.03	0.5	0.3	0.3
Octyl ocanoate	1815	1819	1582	1773	1788	1737	2.17	2.13	2.19	12.9	10.9	14.2
o-Toluidine	735	947	765	1069	1101	1088	3.16	3.73	2.81	65.3	84.8	53.7
PCB 10	1600	1650	1552	1611	1732	1616	3.53	3.55	3.42	76.8	75.3	71.9
PCB 100	2115	2230	2099	2026	2193	2017	3.51	3.77	3.59	73.3	81.4	77.2
PCB 101	2210	2210	2087	2110	2191	2075	3.53	3.82	3.61	74.0	84.3	77.9
PCB 102	2150	2198	2097	2057	2182	2036	3.68	3.79	3.61	81.1	83.4	78.0
PCB 103	2105	2196	2089	2017	2180	2020	3.46	3.78	3.58	71.0	82.8	76.9
PCB 104	2045	2195	2099	1965	2169	1984	3.68	3.76	3.59	81.5	81.1	77.4
PCB 105	2400	2252	2223	2295	2217	2189	3.99	3.84	3.88	93.6	85.3	94.2
PCB 106	2345	2213	2136	2240	2196	2129	3.72	3.81	3.62	81.6	84.6	81.1
PCB 107	2335	2218	2180	2230	2197	2160	3.66	3.83	3.77	78.9	84.9	86.8
PCB 108	2240	2218	2149	2138	2192	2143	3.64	3.79	3.73	78.8	83.2	85.7
PCB 109	2335	2208	2144	2230	2187	2086	3.68	3.80	3.67	79.8	83.8	82.3
PCB 11	1800	1697	1597	1762	1759	1722	3.44	3.60	3.54	71.7	76.3	76.1
PCB 110	2290	2210	2180	2186	2190	2115	3.80	3.83	3.77	85.8	84.8	86.9
PCB 111	2265	2204	2108	2162	2189	2114	3.37	3.79	3.57	66.1	83.1	77.8
PCB 112	2235	2198	2109	2133	2183	2065	3.61	3.80	3.53	77.5	83.9	76.1
PCB 113	2215	2196	2108	2114	2182	2070	3.51	3.79	3.58	73.0	83.1	77.9
PCB 114	2370	2240	2136	2265	2213	2120	3.81	3.83	3.62	85.5	84.9	81.1
PCB 115	2265	2236	2144	2162	2204	2078	3.72	3.82	3.67	82.3	84.2	82.3
PCB 117	2260	2210	2109	2157	2191	2057	3.73	3.81	3.52	82.8	84.2	76.1
PCB 118	2350	2244	2180	2245	2214	2151	3.65	3.84	3.77	78.3	85.2	86.8
PCB 119	2235	2241	2157	2133	2204	2092	3.55	3.81	3.76	74.7	83.4	86.2
PCB 12	1815	1712	1623	1773	1766	1740	3.49	3.62	3.53	74.0	77.3	75.8
PCB 120	2280	2215	2108	2176	2196	2106	3.39	3.80	3.57	66.9	83.4	77.8
PCB 121	2160	2211	2085	2065	2182	2047	3.30	3.76	3.55	63.5	81.2	77.1
PCB 122	2375	2225	2223	2270	2199	2198	3.87	3.83	3.88	88.3	85.0	94.2
PCB 123	2340	2244	2149	2235	2210	2134	3.67	3.81	3.73	79.3	83.5	85.7
PCB 124	2330	2218	2166	2225	2196	2151	3.61	3.82	3.73	76.6	84.5	84.8
PCB 126	2475	2260	2223	2370	2223	2237	3.82	3.85	3.74	85.3	85.5	87.8
PCB 127	2405	2226	2149	2300	2199	2190	3.52	3.80	3.69	71.8	83.3	84.3

Compound	$^{1}t_R$ (s) *			LRI *			$^{2}t_R$ (s) *			PEG-^{2}I *		
	experi-mental	PLS	Chrom Genius	experi-mental	PLS	Chrom Genius	experi-mental	PLS	Chrom Genius	experi-mental	PLS	Chrom Genius
PCB 128	2505	2411	2435	2400	2352	2256	4.18	3.90	4.02	101.9	87.3	93.9
PCB 129	2465	2380	2358	2360	2328	2256	3.97	3.88	3.92	92.3	86.7	89.8
PCB 13	1815	1709	1597	1773	1766	1714	3.50	3.60	3.53	74.5	76.6	75.7
PCB 130	2440	2378	2337	2335	2328	2253	3.82	3.87	3.86	85.5	86.2	87.6
PCB 131	2370	2371	2367	2265	2321	2199	3.90	3.88	3.93	89.7	86.3	90.5
PCB 132	2395	2371	2337	2290	2321	2201	4.05	3.87	3.87	96.4	86.2	87.8
PCB 133	2365	2366	2243	2260	2322	2250	3.52	3.87	3.70	72.1	85.8	81.2
PCB 134	2360	2355	2283	2255	2315	2202	3.87	3.88	3.79	88.4	86.6	84.8
PCB 135	2320	2359	2243	2215	2315	2199	3.70	3.86	3.70	80.9	85.7	81.3
PCB 136	2280	2351	2243	2176	2309	2150	3.98	3.86	3.70	94.2	85.7	81.4
PCB 137	2435	2393	2259	2330	2342	2240	3.77	3.87	3.68	83.2	86.1	80.5
PCB 139	2340	2385	2266	2235	2334	2183	3.71	3.86	3.72	81.2	85.6	82.1
PCB 14	1750	1669	1552	1723	1748	1702	3.27	3.56	3.54	64.0	75.5	75.9
PCB 140	2345	2389	2340	2240	2330	2192	3.76	3.85	3.85	83.4	84.9	87.2
PCB 141	2420	2378	2243	2315	2329	2244	3.72	3.88	3.70	81.0	86.4	81.3
PCB 142	2370	2370	2233	2265	2319	2190	3.90	3.87	3.67	89.7	86.2	80.0
PCB 143	2355	2358	2259	2250	2318	2199	3.87	3.85	3.69	88.5	85.7	80.6
PCB 144	2325	2371	2252	2220	2322	2187	3.66	3.87	3.72	79.0	86.0	82.0
PCB 145	2265	2350	2266	2162	2310	2144	3.86	3.85	3.73	88.8	85.3	82.3
PCB 146	2380	2377	2243	2275	2329	2240	3.55	3.87	3.70	73.4	86.0	81.2
PCB 147	2330	2359	2194	2225	2320	2187	3.69	3.86	3.55	80.3	85.7	75.5
PCB 148	2270	2363	2246	2167	2317	2189	3.47	3.84	3.67	70.7	84.6	80.2
PCB 149	2340	2370	2243	2235	2322	2189	3.73	3.87	3.70	82.1	86.0	81.3
PCB 15	1830	1736	1597	1785	1784	1705	3.55	3.62	3.52	76.7	77.0	75.3
PCB 150	2235	2356	2246	2133	2311	2141	3.71	3.84	3.68	82.1	84.6	80.3
PCB 151	2315	2351	2171	2210	2313	2190	3.63	3.87	3.58	77.7	86.2	76.5
PCB 152	2250	2339	2194	2148	2306	2147	3.82	3.85	3.55	87.1	85.4	75.6
PCB 154	2290	2390	2246	2186	2335	2180	3.49	3.86	3.67	71.4	85.0	80.1
PCB 155	2185	2376	2250	2087	2326	2132	3.47	3.83	3.65	71.3	83.9	79.1
PCB 156	2560	2415	2358	2458	2353	2300	3.89	3.90	3.80	88.3	87.2	89.6
PCB 157	2570	2419	2435	2468	2358	2310	3.98	3.91	3.84	92.5	87.4	93.8
PCB 158	2455	2407	2367	2350	2345	2239	3.80	3.89	3.84	84.5	86.8	90.3
PCB 159	2490	2374	2259	2385	2331	2304	3.57	3.86	3.60	73.7	85.8	80.4
PCB 16	1860	1865	1828	1808	1899	1818	3.78	3.68	3.55	87.2	79.6	75.8
PCB 160	2450	2379	2233	2345	2326	2240	3.81	3.88	3.61	85.0	86.3	79.9
PCB 161	2380	2366	2266	2275	2323	2242	3.50	3.85	3.67	71.1	85.3	82.0
PCB 162	2500	2386	2337	2395	2334	2307	3.64	3.88	3.76	76.9	86.3	87.4
PCB 163	2450	2375	2283	2345	2329	2243	3.77	3.89	3.72	83.1	87.0	84.7
PCB 164	2450	2378	2337	2345	2328	2252	3.78	3.88	3.77	83.6	86.3	87.6
PCB 165	2375	2356	2194	2270	2319	2246	3.45	3.85	3.49	68.8	85.5	75.4
PCB 166	2480	2406	2233	2375	2344	2230	3.91	3.89	3.61	89.5	86.7	79.9
PCB 167	2515	2413	2337	2411	2351	2297	3.66	3.89	3.76	77.8	86.6	87.4
PCB 168	2395	2397	2340	2290	2337	2242	3.57	3.85	3.76	74.2	85.0	87.1
PCB 169	2640	2427	2435	2544	2365	2369	3.79	3.91	4.01	83.3	87.6	93.6
PCB 17	1820	1859	1752	1777	1898	1764	3.54	3.65	3.46	76.3	78.2	73.4
PCB 170	2650	2553	2520	2556	2478	2491	4.08	3.95	4.02	96.6	89.3	93.6
PCB 171	2555	2551	2515	2453	2471	2414	3.98	3.94	4.02	92.5	88.5	93.6
PCB 172	2580	2524	2422	2479	2459	2471	3.71	3.93	3.85	79.9	88.5	86.6

Compound	^1t_R (s) *			LRI *			^2t_R (s) *			PEG-^2I *		
	experi-mental	PLS	Chrom Genius	experi-mental	PLS	Chrom Genius	experi-mental	PLS	Chrom Genius	experi-mental	PLS	Chrom Genius
PCB 173	2565	2522	2460	2463	2454	2416	4.05	3.94	3.95	95.7	89.0	90.6
PCB 175	2480	2512	2414	2375	2448	2395	3.63	3.92	3.84	76.5	87.8	86.4
PCB 176	2435	2505	2414	2330	2442	2332	3.91	3.92	3.85	89.7	87.9	86.5
PCB 177	2545	2518	2447	2442	2454	2411	3.93	3.93	3.91	90.2	88.7	88.9
PCB 178	2465	2503	2351	2360	2445	2391	3.60	3.92	3.74	75.2	88.2	82.1
PCB 18	1820	1847	1762	1777	1894	1778	3.54	3.67	3.47	76.3	79.4	75.8
PCB 180	2595	2551	2422	2495	2476	2458	3.75	3.94	3.85	81.7	88.9	86.6
PCB 181	2535	2540	2385	2432	2470	2373	3.89	3.93	3.75	88.4	88.6	82.8
PCB 182	2485	2538	2431	2380	2467	2384	3.66	3.92	3.82	77.9	87.7	85.4
PCB 183	2495	2539	2414	2390	2466	2383	3.70	3.93	3.84	79.7	88.2	86.4
PCB 184	2390	2534	2419	2285	2459	2312	3.66	3.90	3.81	78.4	86.8	85.1
PCB 185	2520	2526	2340	2416	2457	2378	3.77	3.94	3.73	82.9	88.6	81.8
PCB 186	2455	2505	2385	2350	2445	2323	3.97	3.92	3.76	92.4	88.3	82.9
PCB 187	2485	2514	2351	2380	2452	2379	3.65	3.93	3.74	77.4	88.5	82.1
PCB 188	2375	2500	2360	2270	2446	2309	3.63	3.91	3.70	77.2	87.6	80.7
PCB 189	2715	2560	2520	2628	2484	2561	3.85	3.96	3.92	85.5	89.4	93.5
PCB 190	2655	2557	2460	2561	2478	2466	3.96	3.96	3.89	91.0	89.4	90.5
PCB 191	2605	2558	2515	2506	2477	2477	3.76	3.94	3.94	82.2	88.5	93.5
PCB 192	2580	2521	2385	2479	2458	2447	3.60	3.93	3.73	74.8	88.4	82.7
PCB 193	2595	2525	2447	2495	2460	2473	3.73	3.94	3.85	80.8	88.8	88.8
PCB 194	2785	2702	2647	2706	2618	2659	3.93	4.03	4.02	88.9	92.7	92.8
PCB 195	2740	2695	2661	2656	2611	2590	4.14	4.03	4.06	98.8	92.7	94.4
PCB 196	2680	2691	2633	2589	2610	2578	3.86	4.02	4.03	86.2	92.2	92.4
PCB 197	2585	2685	2620	2484	2598	2502	3.81	4.00	4.04	84.6	91.1	92.5
PCB 198	2660	2660	2557	2567	2591	2568	3.76	4.01	3.97	81.7	92.2	89.6
PCB 199	2670	2665	2570	2578	2592	2574	3.81	4.01	3.96	84.0	92.0	89.8
PCB 2	1540	1482	1456	1569	1595	1533	3.35	3.50	3.43	68.7	73.7	71.1
PCB 20	1955	1875	1801	1888	1907	1848	3.67	3.69	3.55	81.6	79.7	76.5
PCB 200	2615	2653	2557	2517	2585	2502	4.00	4.01	3.97	93.2	92.2	89.8
PCB 201	2565	2652	2557	2463	2584	2498	3.80	4.00	3.97	84.1	91.6	89.5
PCB 202	2550	2651	2495	2447	2578	2494	3.73	4.00	3.88	80.9	91.3	86.1
PCB 204	2570	2683	2566	2468	2601	2481	3.79	3.99	3.90	83.7	91.1	86.7
PCB 205	2790	2702	2661	2712	2617	2660	3.91	4.03	4.00	87.9	92.8	91.9
PCB 208	2735	2809	2713	2650	2720	2677	3.88	4.07	3.99	86.8	94.4	90.1
PCB 21	1950	1875	1766	1883	1905	1824	3.68	3.67	3.52	82.1	79.3	75.4
PCB 22	1970	1887	1801	1900	1915	1843	3.73	3.70	3.55	84.3	80.1	76.5
PCB 23	1885	1859	1724	1829	1899	1797	3.43	3.66	3.47	70.9	79.0	73.2
PCB 24	1840	1851	1724	1792	1892	1768	3.62	3.66	3.47	79.9	78.9	73.4
PCB 25	1915	1868	1724	1854	1905	1795	3.47	3.65	3.46	72.6	78.3	72.8
PCB 26	1915	1857	1745	1854	1902	1807	3.44	3.67	3.47	71.2	79.5	73.2
PCB 27	1840	1848	1724	1792	1891	1771	3.59	3.64	3.46	78.5	78.1	73.0
PCB 29	1895	1870	1724	1838	1905	1792	3.43	3.67	3.47	70.8	79.2	73.2
PCB 3	1555	1498	1456	1579	1605	1540	3.35	3.51	3.43	68.6	74.3	71.9
PCB 30	1780	1856	1700	1746	1893	1749	3.34	3.63	3.43	67.2	77.4	71.8
PCB 31	1935	1869	1745	1871	1909	1802	3.48	3.68	3.47	72.9	79.9	73.2
PCB 32	1865	1860	1724	1813	1899	1765	3.61	3.65	3.46	79.3	78.5	73.0
PCB 33	1955	1886	1801	1888	1914	1843	3.62	3.70	3.55	79.3	80.1	76.5
PCB 34	1885	1856	1724	1829	1898	1800	3.39	3.65	3.46	69.0	78.1	72.8

Compound	$^{1}t_R$ (s) * experimental	$^{1}t_R$ (s) * PLS	$^{1}t_R$ (s) * ChromGenius	LRI * experimental	LRI * PLS	LRI * ChromGenius	$^{2}t_R$ (s) * experimental	$^{2}t_R$ (s) * PLS	$^{2}t_R$ (s) * ChromGenius	PEG-^{2}I * experimental	PEG-^{2}I * PLS	PEG-^{2}I * ChromGenius
PCB 35	2055	1896	1801	1974	1922	1893	3.62	3.70	3.54	78.7	80.3	76.3
PCB 36	1990	1866	1724	1917	1906	1877	3.36	3.65	3.45	67.0	78.3	72.7
PCB 37	2070	1923	1801	1987	1939	1883	3.70	3.72	3.54	82.3	80.7	76.3
PCB 38	2030	1884	1766	1952	1912	1859	3.55	3.68	3.51	75.6	79.5	75.2
PCB 39	2010	1878	1724	1935	1914	1867	3.44	3.66	3.45	70.6	78.6	72.6
PCB 4	1600	1676	1604	1611	1743	1642	3.60	3.58	3.42	80.0	76.0	72.0
PCB 40	2100	2051	2124	2013	2049	2003	3.97	3.77	3.89	94.7	82.6	99.2
PCB 41	2085	2042	2048	2000	2048	1955	3.84	3.74	3.73	88.7	81.9	89.5
PCB 42	2065	2043	2030	1983	2048	1944	3.73	3.74	3.71	83.7	81.4	87.0
PCB 43	2015	2033	1970	1939	2037	1927	3.57	3.73	3.59	76.6	80.8	80.0
PCB 44	2060	2036	2025	1978	2046	1959	3.73	3.76	3.70	83.7	82.5	86.0
PCB 45	1980	2025	1970	1909	2030	1894	3.81	3.72	3.60	87.9	80.8	80.0
PCB 46	2000	2022	2030	1926	2033	1916	3.89	3.73	3.71	91.5	81.1	87.0
PCB 47	2030	2085	1940	1952	2057	1887	3.51	3.71	3.49	73.7	78.7	76.3
PCB 48	2030	2044	1970	1952	2044	1921	3.57	3.74	3.59	76.5	81.1	80.0
PCB 49	2025	2041	1935	1948	2043	1901	3.49	3.72	3.52	72.8	80.4	75.4
PCB 5	1710	1692	1623	1693	1751	1703	3.59	3.60	3.48	79.0	76.7	74.1
PCB 50	1925	2041	1966	1863	2033	1873	3.53	3.70	3.56	75.3	79.1	79.1
PCB 51	1965	2050	1940	1896	2033	1861	3.67	3.69	3.50	81.5	78.4	76.3
PCB 53	1955	2020	1935	1888	2028	1875	3.67	3.71	3.53	81.6	80.2	75.4
PCB 54	1895	2029	1940	1838	2019	1835	3.93	3.68	3.50	94.0	78.1	76.3
PCB 55	2175	2052	2012	2078	2056	1990	3.77	3.75	3.72	85.3	82.0	82.2
PCB 56	2195	2071	2074	2096	2063	2032	3.85	3.78	3.88	88.9	83.0	85.7
PCB 57	2110	2043	1969	2022	2045	1962	3.49	3.73	3.59	72.4	81.0	78.1
PCB 58	2125	2040	1997	2035	2048	1986	3.58	3.73	3.70	76.6	81.2	81.9
PCB 59	2060	2035	1969	1978	2038	1927	3.68	3.73	3.60	81.4	80.9	78.4
PCB 6	1700	1687	1597	1685	1751	1677	3.46	3.59	3.48	73.0	76.1	74.0
PCB 60	2195	2079	2012	2096	2073	1983	3.85	3.76	3.72	88.9	82.4	82.2
PCB 61	2140	2042	1954	2048	2053	1945	3.65	3.74	3.54	79.8	82.3	78.6
PCB 62	2035	2049	1961	1957	2040	1913	3.58	3.73	3.56	76.9	80.6	77.7
PCB 63	2135	2055	1969	2043	2052	1955	3.55	3.74	3.59	75.2	81.2	78.0
PCB 64	2085	2047	1969	2000	2045	1921	3.74	3.74	3.60	84.1	81.2	78.4
PCB 65	2030	2022	1926	1952	2041	1892	3.58	3.74	3.42	77.0	81.9	73.5
PCB 66	2160	2078	1997	2065	2072	1972	3.63	3.76	3.70	78.8	81.9	81.9
PCB 67	2120	2054	1969	2030	2051	1955	3.51	3.74	3.59	73.3	81.2	78.0
PCB 68	2090	2067	1924	2004	2047	1927	3.39	3.70	3.49	67.8	78.4	74.7
PCB 69	2005	2050	1946	1930	2040	1906	3.42	3.71	3.55	69.7	79.1	76.3
PCB 7	1670	1671	1552	1663	1747	1649	3.36	3.56	3.46	68.6	75.5	73.5
PCB 70	2155	2056	2015	2061	2060	1988	3.59	3.77	3.71	77.0	83.0	79.4
PCB 72	2085	2038	1940	2000	2043	1942	3.34	3.72	3.53	65.5	80.3	75.2
PCB 73	2010	2046	1924	1935	2033	1899	3.44	3.69	3.49	70.6	78.1	75.0
PCB 74	2145	2081	1969	2052	2069	1949	3.54	3.76	3.59	74.7	81.6	78.0
PCB 75	2030	2078	1946	1952	2058	1900	3.45	3.72	3.55	71.0	79.6	76.3
PCB 76	2150	2050	2012	2057	2055	1990	3.66	3.75	3.72	80.2	82.1	82.2
PCB 77	2295	2107	2074	2190	2088	2063	3.81	3.80	3.78	86.2	83.6	97.6
PCB 78	2250	2060	2012	2148	2063	2028	3.62	3.76	3.65	77.8	82.2	88.2
PCB 79	2230	2060	1997	2129	2062	2016	3.51	3.75	3.65	72.9	81.7	85.8
PCB 80	2160	2064	1924	2065	2048	1970	3.26	3.70	3.44	61.7	78.3	75.5

Compound	$^{1}t_R$ (s) *			LRI *			$^{2}t_R$ (s) *			PEG-^{2}I *		
	experi-mental	PLS	Chrom Genius	experi-mental	PLS	Chrom Genius	experi-mental	PLS	Chrom Genius	experi-mental	PLS	Chrom Genius
PCB 81	2270	2087	2012	2167	2080	2020	3.75	3.77	3.65	83.7	82.6	88.2
PCB 82	2310	2216	2279	2205	2192	2151	4.06	3.83	4.01	97.7	84.9	94.4
PCB 83	2240	2198	2190	2138	2183	2125	3.75	3.82	3.83	83.9	84.5	86.9
PCB 84	2200	2190	2190	2100	2176	2081	3.98	3.81	3.83	94.9	84.4	87.0
PCB 85	2275	2236	2182	2171	2203	2104	3.84	3.80	3.80	87.8	83.4	85.8
PCB 86	2255	2203	2133	2152	2188	2103	3.78	3.81	3.69	85.2	84.4	81.3
PCB 88	2160	2199	2143	2065	2179	2052	3.75	3.80	3.72	84.4	83.7	82.4
PCB 89	2205	2201	2182	2105	2179	2069	3.97	3.79	3.81	94.4	83.1	86.0
PCB 9	1675	1689	1571	1667	1750	1653	3.35	3.59	3.47	68.1	76.1	73.6
PCB 90	2210	2207	2097	2110	2189	2079	3.54	3.80	3.61	74.5	83.3	77.9
PCB 91	2170	2199	2097	2074	2182	2036	3.76	3.79	3.61	84.8	83.3	78.0
PCB 92	2200	2199	2087	2100	2184	2083	3.49	3.81	3.61	72.2	84.0	77.9
PCB 93	2150	2188	2068	2057	2175	2044	3.75	3.80	3.56	84.4	83.8	76.2
PCB 94	2130	2187	2097	2039	2175	2044	3.67	3.78	3.61	80.7	83.0	78.0
PCB 95	2155	2191	2087	2061	2178	2040	3.74	3.81	3.61	83.9	84.0	78.0
PCB 96	2095	2179	2097	2009	2168	2003	3.95	3.78	3.61	93.8	83.0	78.1
PCB 97	2255	2209	2190	2152	2189	2117	3.77	3.82	3.83	84.7	84.7	86.9
PCB 98	2150	2205	2192	2057	2180	2061	3.68	3.79	3.82	81.1	82.8	86.4
PCB 99	2220	2234	2097	2119	2206	2071	3.56	3.81	3.61	75.3	83.7	77.9
PEG2	535	280	649	957	897	1082	2.90	3.06	3.15	55.2	66.4	116.4
PEG3	990	908	1071	1215	1196	1344	3.36	3.41	3.65	72.8	75.4	124.9
PEG7	2435	2405	2160	2330	2321	2225	4.11	4.10	4.07	99.0	98.3	115.2
PEG8	2695	2683	2489	2606	2607	2500	4.27	4.24	4.23	105.1	103.7	111.2
Prodiamine	2020	2251	2148	1943	2092	2164	2.73	3.30	2.85	37.7	58.1	32.7
Profenofos	2265	2232	2295	2162	2180	2161	3.80	3.79	4.10	86.0	92.5	104.4
Propachlor	1585	1529	1693	1600	1586	1570	3.45	3.38	2.93	73.2	69.5	49.0
Prothiofos	2255	2315	2289	2152	2238	2200	3.53	3.71	3.73	73.6	86.9	80.6
Pyridaphenthion	2535	2576	2496	2432	2487	2477	4.66	4.56	4.68	124.0	123.3	122.7
Pyriproxyfen	2655	2751	2761	2561	2603	2527	4.18	4.35	4.72	101.2	104.3	95.7
sec-Butylbenzene	625	730	633	1008	1015	1011	2.21	2.19	2.22	22.1	21.5	21.6
Tamoxifen	2795	3128	2660	2718	2948	2655	3.72	4.21	4.01	79.1	97.4	81.5
Tebuconazole	2480	2421	2450	2375	2303	2230	3.84	3.77	3.64	86.3	76.2	89.9
Tebufenpyrad	2595	2704	2516	2495	2533	2454	3.50	4.08	3.36	70.2	91.2	76.2
Tefluthrin	1870	1830	2020	1817	1801	2016	2.26	2.07	2.38	16.8	16.0	32.3
Terbufos	1810	1745	1775	1769	1763	1762	3.12	3.11	2.89	56.9	60.8	49.2
Tetrachlorvinfos	2210	2251	2147	2110	2207	2041	3.93	3.81	4.08	92.5	94.1	99.6
Tetradifon	2615	2553	2282	2517	2450	2523	4.50	4.34	4.33	116.4	116.1	108.4
Tinuvin 328	2730	2824	2821	2644	2652	2820	3.36	3.75	3.75	62.8	74.3	82.0
Tocopheryl acetate	3150	3246	3136	3167	3176	2891	2.82	2.92	2.68	34.7	36.7	23.4
Tolylfluanid	2145	2240	2222	2052	2159	2064	3.87	3.83	3.97	90.0	91.0	96.0
Transfluthrin	1970	1969	1977	1900	1903	1936	2.66	2.63	2.83	34.7	37.2	50.5
trans-Nonachlor	2365	2431	2415	2260	2369	2319	3.71	3.79	3.71	80.9	79.2	79.2
Trifluralin	1675	1836	1740	1667	1809	1727	2.24	2.58	2.29	16.7	29.8	25.1
Tri-p-cresyl phosphate	2695	2818	2723	2606	2710	2632	4.22	4.33	4.33	102.8	115.6	106.6
Tris(2.3-dichloropropyl)phosphate	2415	2240	2716	2310	2174	2587	3.71	3.45	4.17	80.5	73.8	107.8
tritriacontane	3245	3271	3493	3300	3301	3454	2.10	2.09	2.08	0.5	0.2	0.3
Vinclozolin	1945	2160	2046	1879	2103	1895	3.33	4.08	3.98	65.9	97.6	93.3

* $^{1}t_{R}$, LRI, $^{2}t_{R}$, and PEG-^{2}I are the first-dimension retention time, linear retention index, second-dimension retention time and polyethylene glycol based second-dimension retention index, respectively.
